# The converse to Bergmann's rule in bumblebees, a phylogenetic approach

**DOI:** 10.1002/ece3.2321

**Published:** 2016-08-02

**Authors:** Víctor Hugo Ramírez‐Delgado, Salomón Sanabria‐Urbán, Martin A. Serrano‐Meneses, Raúl Cueva del Castillo

**Affiliations:** ^1^ Lab. de Ecología; UBIPROFacultad de Estudios Superiores IztacalaUniversidad Nacional Autónoma de MéxicoA.P. 314Tlalnepantla54090 MéxicoMéxico; ^2^ Laboratorio de Biología EvolutivaCentro Tlaxcala de Biología de la ConductaUniversidad Autónoma de TlaxcalaCarretera Tlaxcala‐Puebla km 1.590070 TlaxcalaMéxico; ^3^ Posgrado en Ciencias BiológicasUniversidad Autónoma de TlaxcalaMéxico

**Keywords:** Bergmann's rule, bumblebees, fecundity, life history

## Abstract

Two patterns commonly emerge when animal body size is analyzed as a function of latitudinal distribution. First, body size increases with latitude, a temperature effect known as Bergmann's rule, and second, the converse to Bergmann's rule, a pattern in which body size decreases with latitude. However, other geographic patterns can emerge when the mechanisms that generate Bergmann's and the converse to Bergmann's clines operate together. Here, we use phylogenetic comparative analysis in order to control for phylogenetic inertia, and we show that bumblebees exhibit the converse to Bergmann's rule. Bumblebee taxa are distributed worldwide in temperate and tropical regions. The largest species are found in places with high water availability during the driest time of the year. Nonetheless, large body size is constrained by extreme temperatures. Bumblebees’ body size could be related to a higher extent to the size of food rewards to be harvested than to the energetic advantages of thermoregulation. Moreover, we found that the body size of eusocial and cuckoo species responded in the same way to environmental variables, suggesting that they have not diverged due to different selective pressures.

## Introduction

Body size has profound consequences on fitness, female fecundity, and male mating success, which usually increase with body size (Alcock and Thornhill 1983; Wiklund and Karlsson 1988; Honěk 1993; Andersson 1994). However, body size depends on the regulation of growth rates and development times (Blanckenhorn et al. 2007), and it can be influenced by environmental temperature, season length, productivity, and mortality (Blanckenhorn and Demont 2004; Chown and Gaston 2010). In cooler environments both, ectotherms (Ray 1960; Stevenson 1985; Barlow 1994; Hawkins and Lawton 1995) and endothermic animals (Cushman et al. 1993; Blackburn and Gaston 1994a,b) tend to attain larger adult body sizes. Bergmann (Bergmann 1848) suggested that larger endotherms possess smaller surface‐to‐volume ratios more conducive to conserving heat in cold climates. Thus, body size tends to increase with increasing distance from the equator, a trend referred to as Bergmann's rule (Bergmann 1848; James 1970). Bergmann's rule is associated with a temperature–size rule (Kingsolver and Huey 2008; Horne et al. 2015) that describes the plastic response in which the size of a given animal at maturity is inversely related to the temperature experienced during ontogeny (Atkinson 1994). At high temperatures, growth rates increase, development times are shorter, and adults reach a smaller body size (Atkinson 1994; Sibly and Atkinson 1994; Angilletta and Dunham 2003). In insects and other ectotherms, an increase in temperatures associated with decreasing distance to the equator can explain these patterns. Nonetheless, there is not a consensus regarding their support to Bergmann's rule, as it occurs with endotherms (Angilletta and Sears 2004).

Even though many ectotherms follow Bergmann's rule, other species show the opposite pattern. Some insect species can attain a large adult body size when they grow at high temperatures (Atkinson 1994; Mousseau 1997; Horne et al. 2015). Moreover, shorter seasons toward the poles limit growth and development times, constraining the body size that organisms can achieve (Park 1949; Masaki 1967; Brennan and Fairbairn 1995; Mousseau 1997).

In arthropods, the geographic patterns associated with Bergmann's rule and the converse to Bergmann's rule can be explained by the adaptation of univoltine and multivoltine species to changing season lengths. In multivoltine terrestrial species, body size decreases with increasing temperature and decreasing latitude. Thus, smaller species produce multiple generations annually. On the other hand, univoltine species could take advantage of a longer growing season at lower latitudes by developing a larger adult size, and would therefore exhibit the converse Bergmann's cline (Kozlowski et al. 2004; Horne et al. 2015). Other geographic patterns can emerge when the mechanisms that generate Bergmann's and the converse to Bergmann's clines operate together (Blanckenhorn and Demont 2004; Shelomi 2012). Saw‐tooth clines rise when taxa shift from univoltine to polyvoltine generations according to changes in seasonality (Kivelä et al. 2011). Nonetheless, these patterns are not expected if the counter gradient variation emerges. Populations of a given species compensate seasonal limitations at higher latitudes by evolving faster growth and larger body sizes compared to their low latitude conspecifics (Blanckenhorn and Demont 2004).

Even though several studies in insects and other ectotherms have explored the latitudinal clines associated with body size (see Shelomi 2012 and Horne et al. 2015 for reviews), just few of them have considered the potential phylogenetic effects to separate the historical effects from adaptive trends in the clinal patterns of species (see Traynor and Mayhew 2005 and Swaegers et al. 2014). Using a phylogenetic comparative approach, in this study, we tested Bergmann's rules in bumblebees (*Bombus* sp.) considering both geographic and climatic parameters. Bumblebees are ideal to test Bergmann's rule because they are distributed from the arctic to the tropics and they have thermoregulatory capacities (Goulson 2010). Moreover, reliable phylogenies of the group are available (Cameron et al. 2007), which allow the use of phylogenetic comparative analyses in order to separate historical effects from adaptive trends in this group of insects.

Most bumblebee taxa are eusocial and annual organisms, with few exceptions (Goulson 2010). Fertilized queens emerge from their hibernacula in late winter or early spring and establish new nests for the first generations of workers that will help grow the colonies. Once the queen has established a colony of workers, her main activity is to lay more eggs, while the workers maintain the colony and forage for food (see Alford 1975 for details). After producing the first generation of workers, at some point in the spring or summer, the queen biases her offspring production in favor of new queens and males, which leave the colony after maturation. In geographic areas where the spring is very short, the queen rears only one batch of workers before commencing the production of reproductive individuals. Cuckoo bumblebees, instead, steal a nest from eusocial species: Once a cuckoo female locates a nest, she enters, kills the queen, and takes over the colony. The cuckoo female lays eggs that develop into new females or males (Goulson 2010).

Like other eusocial bees, in bumblebees, the sexes are similar in morphology albeit they differ in size, with females generally being larger than males (Stubblefield and Seger 1994; Gadagkar 1996). Queens tend to be larger than both males and workers, and store large quantities of fat that are consumed during their hibernation (Richards 1946; Cumber 1949; Pereboom 2001). Large size can improve the hibernation capabilities of queens, increase their fecundity, and may grant them an advantage in the competition for nesting places (Owen 1988; Müller and Schmid‐Hempel 1992). Aside from the differences in size and fat storage, the workers are very similar to queens in external morphology. Large individuals can improve the nest's thermoregulation (Bishop and Armbruster 1999) and foraging efficiency (Cnaani and Hefetz 1994; Kapustjanskij et al. 2007). Also, at least in some species, larger males have an advantage in male–male competition (Alcock and Alcock 1983; Amin et al. 2012).

In bumblebees, adult body size depends on the amount of food received (Sutcliffe and Plowright 1988, 1990), although developing queens and males require more food over a longer period, compared to the larvae of workers. Moreover, larvae and pupae incubated under warmer temperatures can grow larger than those developed under lower temperatures (Goulson 2010). However, bumblebees from cold climates are larger than bumblebees from temperate ones, even though the latter are smaller than tropical species (Peat et al. 2005). Perhaps arctic species tend to be larger than southern species due to the thermoregulatory advantages of large body size (Bishop and Armbruster 1999; Heinrich 2004). Nonetheless, in eusocial species, the body size of bumblebees is constrained by the size of the colonies (Cueva del Castillo et al. 2015). Body size could be related to a higher extent to the size of food rewards to be harvested, than to the energetic advantages of thermoregulation (Heinrich 1993).

Despite the fact that bumblebees have thermoregulatory capacities, we expected that these taxa would follow the converse to Bergmann's rule because they are univoltine and because the impact of the size on food rewards to be harvested can be stronger than the energetic advantages of thermoregulation. Bumblebees could take advantage of a longer growing season at lower latitudes by developing to a larger adult size. Moreover, because in social species, queens and males represent the reproductive success of the colonies and these are also the most energetically demanding individuals, we expected that the converse to Bergmann's rule would be stronger on them than on workers and females and males of cuckoo species. In addition to differences among social bumblebees, we expected differences in body size between social and cuckoo bumblebees.

As a rule of thumb, in eusocial insects, there is an extreme bias in body size sexual dimorphism toward reproductive females. This could be explained by selection acting strongly on the fertility of queens and by males being unable to monopolize groups of females (Stubblefield and Seger 1994; Boomsma et al. 2005). Due to the different selective pressures associated with eusocial and cuckoo species with a common evolutionary history, we predicted that queens would be larger than cuckoo females because generally in social insects there is just one reproductive female in the colony, and selection on fecundity can be stronger than in females of cuckoo species. Moreover, we expected cuckoo females to be larger than workers because the latter are sterile (see Cueva del Castillo and Fairbairn 2012). On the other hand, because males from social and cuckoo species can be under similar natural and sexual selection pressures, we did not expect significant differences on body size among them.

## Material and Methods

### Morphologic information

In this study, we used Thorax Width as an index of bumblebee body size. We obtained Thorax Width measurements from *Bombus* specimens housed in the Museums of Entomology of the University of California at Riverside and Berkeley, the Natural History Museum of Los Angeles (NHM‐LA), the California Academy of Sciences (CAS), and the Natural History Museum of Paris (NHM‐P). In addition, we performed a bibliographic search for information on the body size of certain *Bombus* species. Most of the body size measurements were retrieved from published papers (Laroca 1972; Inoue and Yokoyama 2006; Inoue et al. 2008; Cueva del Castillo and Fairbairn 2012; Cueva del Castillo et al. 2015), but we also incorporate unpublished measurements for twenty species, including 12 cuckoo bumblebees. In these cases, measurements were taken considering the methods reported by Cueva del Castillo & Fairbairn (Cueva del Castillo and Fairbairn 2012). In total, we obtained the body size measurements from 91 *Bombus* taxa (see Table S1), which involved most of the clades identified in the phylogeny of genus (Cameron et al. 2007). Because specimens were stored under different conditions, inaccuracy in size differences is possible due to differences in preservation conditions among specimens. However, we expected that interspecific differences associated with geographic distribution would be higher than other sources of variation.

### Geographic and climatic information

Temperature and food availability can affect adult body size of bumblebees. In this study, we incorporated temperature parameters associated with the sampling localities of the reviewed museum specimens and the published and unpublished sources. Similarly, as moisture affects flowering (Rathcke and Lacey 1985), we used precipitation parameters as indicators of food availability, as in other comparative studies (Yom‐Tov and Geffen 2006; Branson 2008; Cueva del Castillo et al. 2015). We used Google Earth 7.1.5 (Google Inc 2014) to obtain the geographic coordinates and BioClim (Hijmans et al. 2005) to obtain 14 climatic parameters associated with temperatures and precipitation of the sampling localities. Because bumblebees have an extensive geographic distribution, we estimated mean values of geographic coordinates and the climatic parameters per species considering the geographic and climatic information of all individuals within the taxa (see Table S1). All values were log‐transformed for posterior analysis.

### Phylogeny

From GenBank, we retrieved the available DNA sequences for the 91 studied taxa of *Bombus* and five outgroup taxa, including *B. waltoni*,* Geniotrigona thoracica*,* Heterotrigona itama*,* Trigona amazonensis,* and *Eulaema boliviensis*. The retrieved genetic information comprised partial sequences of the mitochondrial 16 Subunit of ribosomal RNA (16S) and four protein‐encoding nuclear genes: Arginine Kinase (ArgK), Elongation Factor‐1 Alpha F2 copy (EF‐1*α*), Long‐Wavelength Rhodopsin copy 1 (Opsin), and Phosphoenolpyruvate Carboxykinase (PEPCK). The GenBank accession numbers of the used sequences are shown in Table S2. We aligned the sequence of each locus with MUSCLE (Edgar 2004), using default parameters. We excluded nucleotides of uncertain position that were identified in the 16S and PEPCK alignments. ArgK, Opsin, and PEPCK sequences of some outgroup taxa and *Bombus* species contained fragments that were problematic to align, because they were apparently unrelated to the sequence of the other species at the corresponding positions. We treated these highly divergent regions as missing data, as in (Kawakita et al. 2003) and (Cameron et al. 2007), and excluded from the final alignments the single nucleotide insertions that were identified. Following the methods of Simons & Ocheterena (Simmons and Ochoterena 2000), we coded as separate binary characters the gap regions of unambiguous alignment in all sequences alignments, as in previous phylogenetic studies in *Bombus* (Simmons and Ochoterena 2000; Kawakita et al. 2003). The final alignments comprised the following number of aligned positions/gap‐coded characters: 16S, 465/2; ArgK, 876/18; EF‐1*α*, 745/12; Opsin, 690/9; and PEPCK, 987/23. The combined dataset included 3763 nucleotide aligned positions and 64 gap‐coded characters (available at TreeBASE under submission 18924).

We estimated the best partition scheme and models of nucleotide substitution for this combined dataset, using the greedy algorithm implemented in PARTITIONFINDER v. 1.1.1. (Lanfear et al. 2014). The final combined dataset was partitioned into 10 partitions (P1‐P10), which corresponded to the 16S locus (P1), the exon (P2, P4, P6, P8) and intron (P3, P5, P7, P9) regions of each coding loci (ArgK, EF‐1*α*, Opsin, and PEPCK, respectively) and all gap‐coded characters (P10). We individually applied the following substitution models to each partition: GTR+I+G for P1; GTR+G for P3, P7, and P9; JC+I+G for P4; JC+G for P2, P6, and P8; HKY+G for P5; and Standard+G for P10.

**Figure 1:**
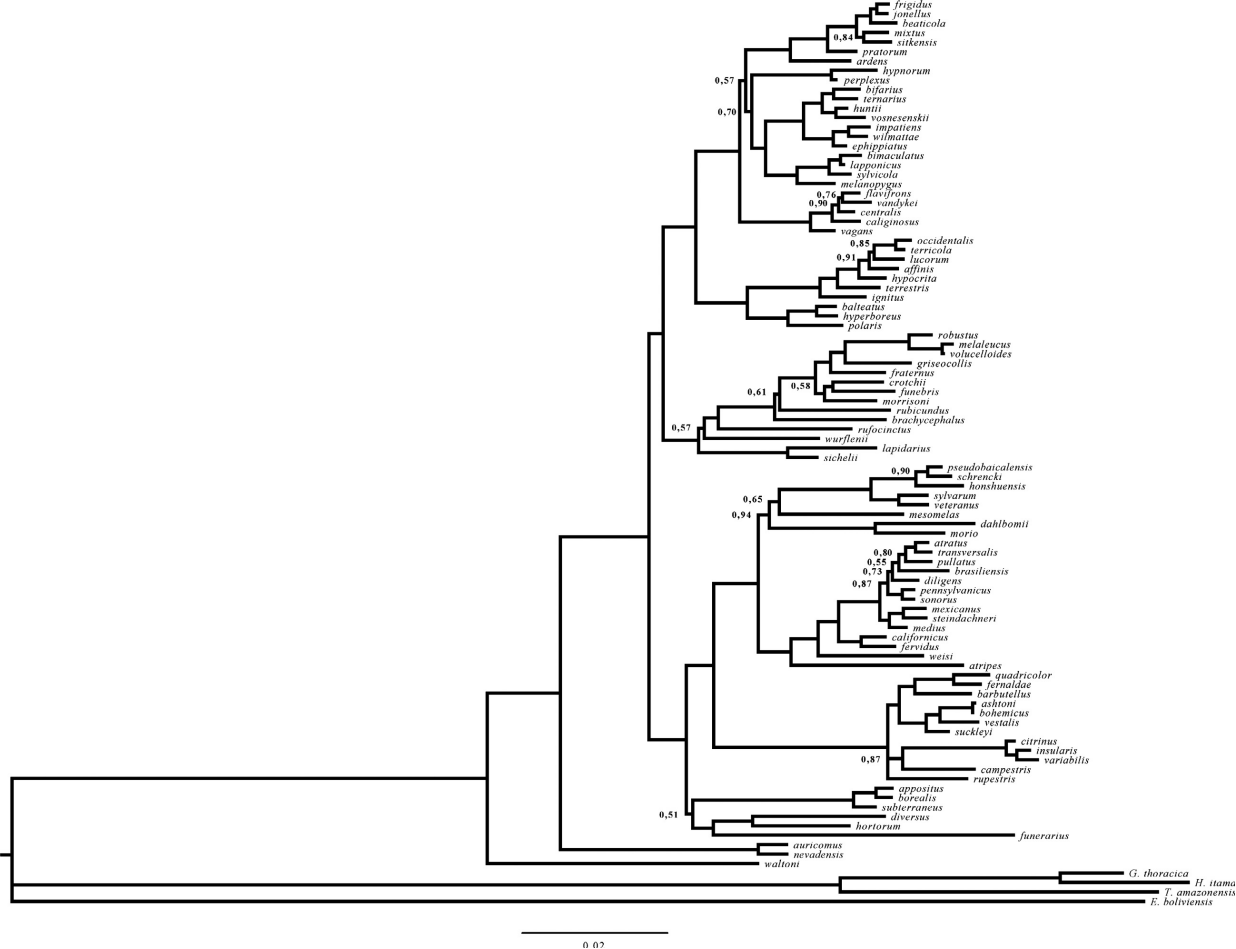
Consensus phylogeny based on a Bayesian analysis of nucleotide sequences of five loci (16S, ArgK, EF‐1*α*, Opsin, and PEPCK) of the 91 studied *Bombus* species and five outgroup taxa (*B. waltoni*,* Geniotrigona thoracica*,* Heterotrigona itama*,* Trigona amazonensis*, and *Eulaema boliviensis*). Values behind the nodes indicate posterior probability values. Only values lower than 0.95 are shown, otherwise these values were higher than 0.95.

We conducted a concatenated Bayesian inference analysis in MRBAYES v. 3.2.6 (Ronquist et al. 2012) of the final combined dataset, applying to each partition the substitution models indicated above. This analysis consisted of four independent runs, each one with 10,000,000 generations and four chains and sampling parameters every 1000 generations. We used the default priors for the other parameters in the analysis. We used TRACER v 1.6 (Rambaut and Drummond 2013) to assess the convergence of parameters and the pertinence of mixing our independent runs. We discarded 25% of the samples obtained prior to stability (burn‐in) and constructed a final consensus tree (Fig. 1), which is also available TreeBASE (submission number 18924). The topology of the resulting consensus tree was largely consistent with the *Bombus* phylogeny (Cameron et al. 2007).

### Comparative analyses

#### Bergmann's rule

We used generalized linear mixed models (GLMM) using Markov chain Monte Carlo algorithms (MCMC) (Hadfield 2010) to test (1) Bergmann's rule and (2) the effect of climatic parameters on the body size of bumblebee taxa. The models were fitted as implemented in the R (R Core Team 2015) package ‘MCMCglmm’ (Hadfield 2010). The package makes use of GLMMs while it marginalizes the random effects in a robust manner. As our goal was to incorporate phylogenetic effects to the models, we constructed a vector containing a tree topology which was associated with the inverse relationship matrix A^−1^. The matrix is formed by assigning the tree topology to the ‘pedigree’ argument of MCMCglmm (Hadfield 2010). The method has been used in a variety of studies, such as to test Darwin's naturalization hypothesis in plants (Sol et al. 2014) and to test the effect of elevation and climatic variables on the body sizes of Neotropical grasshoppers (Sanabria‐Urbán et al. 2015). First, in order to test the effect of latitude on the body size of bumblebees (Bergmann's rule), we generated a dummy variable to incorporate in the analyses data on queens, males, workers, and females and males of cuckoo bumblebees. We then used Thorax Width as the dependent variable, and the dummy variable and latitude as independent variables, and a vector containing the concatenated phylogenetic tree (Fig. 1) as a random variable. Note that by including the tree topology as a random variable, we were able to account for the phylogenetic nonindependence of species (Harvey and Pagel 1991). The models fitted a univariate normal response. The full, saturated models included the first‐order interaction between the dummy variable and latitude, and as no interaction term was statistically significant, they were removed from the models.

#### Climatic variables and body size

To test the effect of climatic variables on the body sizes of bumblebees, we included a dummy variable to incorporate male and female castes of social species and females and males of cuckoo bumblebees. In the phylogenetically corrected GLMMs (see above), we used Thorax Width as the dependent variable, and the dummy variable and climatic parameters (see Table S1) as independent variables. The models fitted a univariate normal response.

All models were run for 5,500,000 iterations after a burn‐in of 1000 iterations and a thinning interval of 500 iterations. The proportion of the total variance in the models was accounted for by the random variable tree topology, which was calculated for each model. We ensured that effective sampling sizes (ESS) were adequate for each model (>10,000). The significance of the predictors was determined when the 95% credible intervals of the effect size excluded zero (e.g. McLean et al. 2013). Finally, for each model, we also determined the extent of the phylogenetic signal by calculating Pagel's *λ* (Pagel 1999).

## Results

### Bergmann's rule

**Figure 2:**
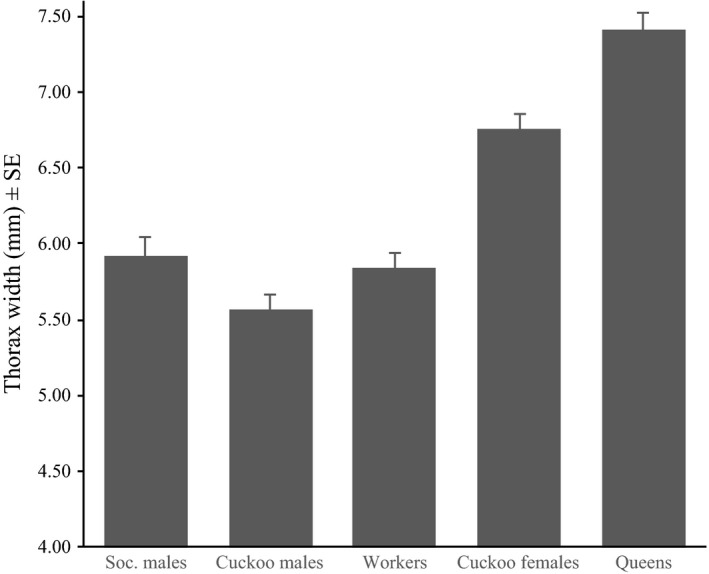
Mean ± SE Thorax Width of females and males from eusocial and cuckoo bumblebee species.

**Figure 3:**
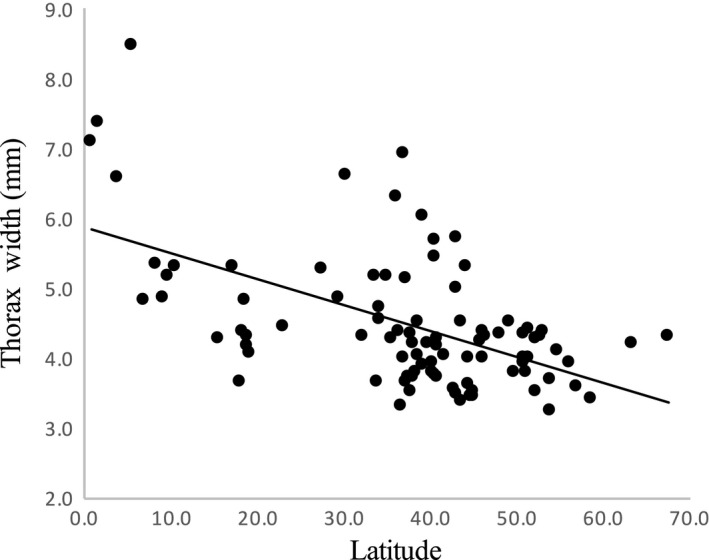
Thorax Width as a function of latitude for 91 bumblebee species. Ordinary least squares regressions fitted are shown for illustrative purposes. Bumblebees exhibit the converse to Bergmann's rule: The largest species are found in lower latitudes.

After controlling for phylogenetic nonindependence among bumblebee taxa, the results of the MCMCglmm analysis indicated significant differences in bumblebees’ body size (Dummy variable; Table 1, Fig. 2). Nonetheless, there were no differences between queens and cuckoo females (*P* MCMC = 0.488), between workers and cuckoo females (*P* MCMC = 0.083), and between eusocial and cuckoo males (*P* MCMC = 0.635). Moreover, according to the inverse Bergmann's rule, we found a negative and significant relationship between latitude and Thorax Width (Fig. 3). The interactions between the dummy variable and all other independent variables were not significant (data not shown in simplified models), indicating a similar body size response to latitude between females and males of eusocial and cuckoo bumblebees. The model showed high *λ* values (*λ* > 0.980), indicating a strong phylogenetic effect on the relationships between the latitude and body size.

**Table 1:** Generalized linear mixed model using Markov chain Monte Carlo algorithms for Thorax Width on Dummy Variable and Latitude distribution of bumblebee taxa

Source	Mean	Lower CI	Upper CI	ESS	*P* MCMC
Intercept	0.631	0.515	0.749	10,998	<0.0001
Dummy variable	−0.076	−0.115	−0.034	10,998	<0.0001
Latitude	0.074	0.064	0.082	12,565	<0.0001

CI, 95% confidence interval; ESS, Estimated sample size; *P* MCMC, Posterior probabilities values of Generalized linear mixed models using Markov chain Monte Carlo algorithms.

### Climatic variables and body size

As the previous models, the results of the MCMCglmm analysis indicated significant differences between bumblebees’ body size for thorax width (Table 2). Even though the dummy variable indicated significant differences in body size, there were no differences between queens and cuckoo females (*P* MCMC = 0.359), between workers and cuckoo females (*P* MCMC = 0.120), and between eusocial and cuckoo males (*P* MCMC = 0.533). In addition, bumblebee body size was significantly and positively related with the precipitation of the driest month of the year, and precipitation seasonality, and negatively related to annual temperature range (Table 2). The other climatic parameters were not significant (data not shown in simplified models). Precipitation seasonality is a measure of the variation in monthly precipitation totals over the course of the year. This index is the ratio of the standard deviation of the monthly total precipitation to the mean monthly total precipitation, whereas annual temperature range is a measure of variation in temperature over a given period and is calculated by subtracting minimum temperature of coldest month from maximum temperature of warmest month. This information is useful when examining whether the distribution of species are affected by ranges of extreme temperatures (O'Donnell and Ignizio 2012). The model showed high *λ* values (*λ* > 0.980), indicating a strong phylogenetic effect on the relationships between the climatic variables and body size.

**Table 2:** Generalized linear mixed model using Markov chain Monte Carlo algorithms. Model for Thorax Width on dummy variable and climatic parameters associated to bumblebee taxa distribution

Source	Mean	Lower CI	Upper CI	ESS	*P* MCMC
Intercept	0.530	0.314	0.750	10,998	<0.0001
Dummy variable	0.073	0.064	0.082	10,998	<0.0001
ATR	−0.152	−0.241	−0.068	11,366	0.0007
PDM	0.058	0.025	0.094	10,998	<0.0001
PS	0.081	0.018	0.143	10,998	0.013

CI, 95% confidence interval; ESS, estimated sample size; *P* MCMC, posterior probability values (ATR; annual temperature range, PDM; precipitation of the driest month of the year, PS; precipitation seasonality).

## Discussion

Despite the fact that bumblebees from cold climates are larger than bumblebees from temperate ones, the geographic variation in *Bombus*’ body size indicated that this group follows the converse to Bergmann's rule: a decrease in body size with latitude (see Peat et al. 2005). Due to the different selective pressures associated with life cycles of eusocial and cuckoo species, we expected differences between them. However, their body sizes responded in the same way to the environmental conditions. The largest bumblebee species can be found in tropical places with high precipitation during the driest time of the year and places with a high seasonality in the rains. On the other hand, the results also suggest that the evolution of large body size is constrained in places with annual extreme temperatures.

In arthropods, the geographic patterns associated with Bergmann's rule and the converse pattern can be explained by the adaptation of univoltine and multivoltine species to changing season lengths. In low and warm latitudes, natural selection favors small multivoltine terrestrial arthropods, which can produce multiple generations in a year. On the other hand, the converse Bergmann's cline is associated with univoltine species (Kozlowski et al. 2004; Horne et al. 2015), as most bumblebees. Toward the poles, shorter seasons limit growth and development times, which constrain the body size that the adults can attain (Park 1949; Masaki 1967; Brennan and Fairbairn 1995; Mousseau 1997), whereas at lower latitudes, univoltine insects can take advantage of a longer growing season by developing a larger adult size that increases both females’ fecundity and male mating success. Despite the fact that some insect species, including bumblebees, can attain a large adult body size when they grow at high temperatures (Atkinson 1994; Mousseau 1997; Couvillon and Dornhaus 2009; Goulson 2010; Horne et al. 2015), the results do not suggest a positive effect of climatic temperatures on body size, even though we previously found a positive effect of precipitation during the warmest trimester of the year on the body size of males (for a smaller number of eusocial species) (Cueva del Castillo et al. 2015).

In bumblebees, adult body size is dependent on the amount of food received (Sutcliffe and Plowright 1988, 1990). Thus, their body size could be related to a higher extent to the size of food rewards to be harvested, than to the energetic advantages of thermoregulation (Heinrich 1993). Because the phenology of bumblebees is strongly related to flower phenology (Pyke et al. 2011), the food availability for bumblebees can be larger in the tropics than in temperate regions. In temperate regions, frost in spring and autumn may limit the flowering season. On the other hand, in tropical forests, many species flower more than once a year (Croat 1975; Opler et al. 1976; Putz 1979). Moreover, in neotropics, many herbs and shrubs flower in the rainy season (Croat 1975; Monasterio and Sarmiento 1976), and tree species flower in the dry and the rainy season (Frankie et al. 1974). Nevertheless, because of the same reason, the bumblebee species with the largest colonies can be found in the tropics. Because both large colonies and large body sizes depend heavily on a large amount of food resources, the canalization of food resources to growing a colony and to body size of colony members generates a trade‐off between body size and colony size, which can constrain the evolution of body size (Cueva del Castillo et al. 2015). Interestingly, despite this potential constraint, larger bumblebee species are found in lower latitudes. As a rule of thumb, in eusocial insects, there is an extreme bias in body size sexual dimorphism toward reproductive females. This could be explained by strong selection acting on the fertility of queens, while males are unable to monopolize groups of females (Stubblefield and Seger 1994; Boomsma et al. 2005). However, this hypothesis was never formally tested before. After controlling for phylogenetic and climatic effects, the body size of queen and females of cuckoo bumblebees is similar, suggesting that their body sizes have not diverged due to differences in selective pressures. Moreover, despite selection on fecundity, the body size of cuckoo females was not different from the size of sterile workers and males from both eusocial and cuckoo species. Neither has diverged, which may suggest similar natural and sexual selection pressures acting among them.

## Conflict of Interest

None declared.

## Data Accessibility

The GenBank accession numbers of the used sequences are shown in supplementary material Table S2 and the final alignment used and the resulting phylogeny are available at TreeBASE under submission 18924.

## Supporting information


**Table S1.** Mean values of Thorax Width, geographic coordinates and the climatic parameters per species, considering the geographic and climatic information of all individuals within the taxa.

**Table S2.** GenBank accession numbers for the *Bombus* taxa and outgroups considered to build the phylogeny used for comparative analysis (see Cameron et al. 2007).
