# Supplementary material for: The converse to Bergmann's rule in bumblebees, a phylogenetic approach
Source: Ecol Evol. 2016 Aug 2;6(17):6160–9. doi: 10.1002/ece3.2321 (PMC5016639; doi:10.1002/ece3.2321)
Supplement: Supplementary file 1 — Table S1. Mean values of Thorax Width, geographic coordinates and the climatic parameters per species, considering the geographic and climatic information of all individuals within the taxa. [file ECE3-6-6160-s001.docx]

| Table S1. Thorax Width (TW; mm) and climatic parameters of social *Bombus* taxa considered in the comparative analyses, Temperature Parameters: ^o^C, Precipitation Parameters: mm (LAT: Latitude, TAR: Temperature Annual Range, MTWQ1: Mean Temperature of the Wettest Quarter of the year, MTW: Maximum Temperature of the Warmest Month of the year, MTWQ2: Mean Temperature of the Warmest Quarter of the year, MTDQ: Mean Temperature of the Driest Quarter of the year, AMT: Annual Mean Temperature, TS: Temperature Seasonality (Coefficient of Variation), AP: Annual Precipitation, PWM: Precipitation of the Wettest Month of the year, PDM: Precipitation of the Driest Month of the year, PWQ1: Precipitation of the Wettest Quarter of the year, PDQ: Precipitation of the Driest Quarter of the year, PWQ2: Precipitation of the Warmest Quarter of the year, PS: Precipitation Seasonality (Coefficient of Variation)). | | | | | | | | | | | | | | | | | | |
| --- | --- | --- | --- | --- | --- | --- | --- | --- | --- | --- | --- | --- | --- | --- | --- | --- | --- | --- |
| *Species* | Sex and Caste | n | TW | LAT | TAR | MTWQ1 | MTW | MTWQ2 | MTDQ | AMT | TS | AP | PWM | PDM | PWQ1 | PDQ | PWQ2 | PS |
| *affinis* | Worker | 5 | 3.51 | 40.09 | 37.50 | 19.32 | 28.22 | 21.08 | -2.70 | 9.70 | 918.56 | 1120.4 | 119.2 | 61.2 | 342.0 | 206.8 | 340.2 | 22.8 |
| *affinis* | Queen | 5 | 5.54 | 39.86 | 35.22 | 11.44 | 27.42 | 20.36 | 3.34 | 9.72 | 842.20 | 1182.6 | 115.2 | 82.0 | 325.4 | 258.6 | 304.0 | 10.0 |
| *affinis* | Male | 3 | 3.77 | 42.18 | 39.37 | 21.00 | 28.63 | 21.00 | -4.03 | 8.83 | 980.50 | 899.0 | 100.7 | 37.0 | 293.7 | 136.3 | 293.7 | 27.7 |
| *appositus* | Worker | 5 | 3.85 | 39.19 | 42.16 | 1.16 | 25.22 | 19.98 | 8.42 | 3.40 | 830.52 | 472.0 | 52.6 | 23.6 | 146.2 | 87.2 | 110.6 | 21.2 |
| *appositus* | Queen | 5 | 5.18 | 39.60 | 34.62 | -0.52 | 22.20 | 11.90 | 10.40 | 2.84 | 683.20 | 554.4 | 76.4 | 20.0 | 210.4 | 70.6 | 82.6 | 40.0 |
| *appositus* | Male | 5 | 3.59 | 39.64 | 39.96 | 7.14 | 25.60 | 20.80 | 2.06 | 4.40 | 799.24 | 458.8 | 68.4 | 13.8 | 184.2 | 52.0 | 148.0 | 47.6 |
| *ardens* | Worker | 4 | 3.79 | 35.32 | 29.40 | 22.40 | 30.40 | 24.90 | 6.60 | 15.70 | 715.40 | 1615.0 | 215.0 | 58.0 | 536.0 | 188.0 | 497.0 | 38.0 |
| *ardens* | Queen | 3 | 5.30 | 35.32 | 29.40 | 22.40 | 30.40 | 24.90 | 6.60 | 15.70 | 715.40 | 1615.0 | 215.0 | 58.0 | 536.0 | 188.0 | 497.0 | 38.0 |
| *ardens* | Male | 2 | 3.76 | 35.32 | 29.40 | 22.40 | 30.40 | 24.90 | 6.60 | 15.70 | 715.40 | 1615.0 | 215.0 | 58.0 | 536.0 | 188.0 | 497.0 | 38.0 |
| *atratus* | Worker | 4 | 3.92 | 14.03 | 14.60 | 21.20 | 28.40 | 22.20 | 20.80 | 21.30 | 75.20 | 1865.0 | 357.0 | 4.0 | 928.0 | 23.0 | 264.0 | 80.0 |
| *atratus* | Queen | 4 | 5.66 | 20.18 | 18.78 | 22.63 | 29.33 | 23.05 | 18.30 | 20.33 | 230.25 | 1333.8 | 258.8 | 4.3 | 696.8 | 46.3 | 346.8 | 85.5 |
| *atratus* | Male | 2 | 3.53 | 20.37 | 19.65 | 22.45 | 29.60 | 23.30 | 17.10 | 20.30 | 253.70 | 1367.5 | 270.0 | 5.5 | 700.5 | 26.0 | 366.5 | 82.0 |
| *atripes* | Worker | 2 | 4.90 | 27.40 | 30.20 | 22.20 | 33.35 | 27.45 | 12.80 | 18.70 | 727.95 | 1803.0 | 324.5 | 47.0 | 897.5 | 171.5 | 587.0 | 63.5 |
| *atripes* | Queen | 5 | 6.24 | 27.34 | 30.14 | 22.26 | 33.38 | 27.42 | 11.66 | 18.76 | 722.34 | 1795.8 | 324.8 | 46.4 | 895.4 | 169.4 | 585.8 | 63.8 |
| *atripes* | Male | 5 | 4.66 | 27.49 | 29.64 | 21.34 | 32.22 | 2662 | 12.46 | 17.88 | 723.96 | 1865.8 | 329.4 | 48.2 | 910.4 | 177.0 | 613.8 | 61.8 |
| *auricomus* | Worker | 5 | 4.76 | 40.00 | 40.50 | 18.24 | 31.06 | 23.26 | 1.10 | 11.10 | 980.54 | 922.8 | 120.2 | 31.8 | 300.8 | 110.2 | 308.8 | 44.0 |
| *auricomus* | Queen | 5 | 6.56 | 39.59 | 40.34 | 20.40 | 31.34 | 23.24 | -1.46 | 11.66 | 976.54 | 960.0 | 123.8 | 32.0 | 306.6 | 113.4 | 317.6 | 40.0 |
| *auricomus* | Male | 4 | 5.71 | 41.98 | 41.83 | 21.45 | 29.55 | 2200 | -5.08 | 9.13 | 1045.03 | 891.5 | 111.5 | 26.8 | 278.5 | 97.8 | 322.8 | 40.8 |
| *balteatus* | Worker | 5 | 3.69 | 70.75 | 40.50 | 3.30 | 9.20 | 4.20 | -26.40 | -11.70 | 1231.00 | 131.0 | 29.0 | 5.0 | 71.0 | 15.0 | 63.0 | 74.0 |
| *balteatus* | Queen | 5 | 5.76 | 57.48 | 35.22 | -1.02 | 10.84 | 4.60 | -15.04 | -7.98 | 956.48 | 366.6 | 59.8 | 13.0 | 158.2 | 46.2 | 81.8 | 63.2 |
| *balteatus* | Male | 4 | 3.20 | 61.81 | 51.85 | 8.85 | 17.43 | 8.95 | -12.15 | -9.43 | 1395.03 | 271.0 | 48.5 | 11.8 | 105.0 | 45.3 | 102.5 | 64.8 |
| *beaticola* | Worker | 125 | 4.00 | 38.41 | 33.16 | 18.58 | 24.87 | 18.58 | -3.46 | 7.35 | 883.92 | 1602.9 | 211.8 | 84.1 | 597.8 | 277.6 | 597.8 | 32.6 |
| *beaticola* | Queen | 1 | 5.40 | 38.42 | 33.10 | 18.40 | 24.70 | 18.40 | -3.70 | 7.20 | 882.20 | 1604.0 | 212.0 | 83.0 | 601.0 | 274.0 | 601.0 | 33.0 |
| *beaticola* | Male | 18 | 4.20 | 38.38 | 33.22 | 18.56 | 24.87 | 18.56 | -3.46 | 7.30 | 885.57 | 1610.0 | 212.8 | 85.1 | 599.6 | 280.6 | 599.6 | 32.3 |
| *bifarius* | Worker | 5 | 2.95 | 47.84 | 34.92 | -9.60 | 28.82 | 18.58 | 18.10 | 8.38 | 786.04 | 519.6 | 72.2 | 19.6 | 206.8 | 68.4 | 81.2 | 38.8 |
| *bifarius* | Queen | 5 | 4.36 | 39.13 | 33.34 | -3.60 | 22.00 | 15.00 | 15.50 | 6.84 | 648.44 | 549.6 | 88.0 | 10.2 | 241.0 | 40.2 | 46.2 | 56.2 |
| *bifarius* | Male | 5 | 2.84 | 43.16 | 26.58 | -1.26 | 19.42 | 11.60 | 11.48 | 4.14 | 556.70 | 1019.8 | 171.8 | 20.2 | 482.6 | 77.6 | 83.4 | 64.6 |
| *bimaculatus* | Worker | 5 | 3.38 | 42.03 | 39.92 | 21.52 | 28.76 | 21.52 | -4.74 | 9.08 | 1013.16 | 924.4 | 107.2 | 34.8 | 306.6 | 132.0 | 306.6 | 30.6 |
| *bimaculatus* | Queen | 5 | 5.04 | 38.95 | 37.32 | 15.80 | 30.04 | 22.94 | 4.56 | 11.68 | 912.50 | 1091.2 | 125.4 | 52.2 | 346.2 | 182.4 | 308.0 | 25.8 |
| *bimaculatus* | Male | 5 | 3.42 | 39.73 | 37.22 | 18.18 | 30.36 | 23.16 | 6.86 | 11.76 | 919.10 | 1022.4 | 110.2 | 58.0 | 311.8 | 196.8 | 284.2 | 19.6 |
| *borealis* | Worker | 5 | 3.94 | 45.40 | 46.78 | 19.56 | 27.66 | 19.56 | -10.90 | 5.34 | 1175.58 | 733.4 | 107.6 | 17.2 | 303.0 | 62.8 | 303.0 | 51.6 |
| *borealis* | Queen | 5 | 4.78 | 46.00 | 47.98 | 19.56 | 27.76 | 19.56 | -11.88 | 4.94 | 1215.08 | 654.2 | 102.2 | 15.4 | 279.8 | 55.0 | 279.8 | 54.4 |
| *borealis* | Male | 2 | 4.00 | 45.50 | 45.15 | 9.95 | 26.75 | 18.45 | -9.10 | 5.05 | 1087.35 | 933.0 | 113.0 | 49.5 | 317.0 | 160.5 | 295.0 | 31.5 |
| *brachycephalus* | Worker | 4 | 4.66 | 4.07 | 15.18 | 21.75 | 27.93 | 2218 | 19.60 | 20.48 | 164.80 | 1145.3 | 182.8 | 29.5 | 510.3 | 107.0 | 330.8 | 49.3 |
| *brachycephalus* | Queen | 9 | 6.11 | 8.66 | 16.97 | 22.67 | 29.63 | 23.30 | 19.60 | 21.27 | 192.80 | 1789.0 | 310.7 | 40.3 | 892.3 | 132.0 | 468.7 | 64.7 |
| *brachycephalus* | Male | 2 | 5.30 | 11.71 | 14.10 | 21.20 | 26.90 | 21.50 | 19.60 | 20.00 | 148.00 | 759.0 | 106.0 | 23.0 | 281.0 | 92.0 | 248.0 | 40.0 |
| *brasilensis* | Worker | 1 | 5.59 | 39.94 | 12.70 | 23.60 | 29.30 | 24.00 | 22.70 | 22.60 | 135.80 | 514.0 | 134.0 | 3.0 | 332.0 | 11.0 | 124.0 | 101.0 |
| *brasilensis* | Queen | 4 | 8.86 | 20.20 | 15.30 | 25.70 | 31.60 | 26.35 | 21.85 | 24.00 | 179.18 | 1232.8 | 199.3 | 32.0 | 556.8 | 122.5 | 412.0 | 55.8 |
| *brasilensis* | Male | 3 | 5.39 | 29.86 | 17.27 | 22.03 | 30.60 | 24.63 | 19.80 | 21.83 | 234.27 | 1179.0 | 177.0 | 47.7 | 462.7 | 153.0 | 359.7 | 57.7 |
| *californicus* | Worker | 5 | 4.00 | 37.03 | 23.48 | 10.04 | 27.72 | 1984 | 19.40 | 14.78 | 393.80 | 609.4 | 125.2 | 1.2 | 326.0 | 8.0 | 23.8 | 88.6 |
| *californicus* | Queen | 5 | 5.53 | 37.16 | 28.72 | 8.52 | 30.86 | 21.36 | 21.16 | 14.64 | 522.96 | 606.4 | 115.8 | 1.6 | 312.6 | 8.4 | 14.2 | 85.4 |
| *californicus* | Male | 5 | 3.54 | 38.29 | 31.30 | 7.02 | 32.22 | 21.64 | 21.46 | 13.48 | 602.52 | 645.2 | 119.4 | 2.0 | 325.4 | 11.8 | 16.0 | 82.6 |
| *caliginosus* | Worker | 5 | 3.18 | 36.43 | 18.04 | 10.46 | 22.54 | 16.08 | 15.30 | 13.02 | 231.34 | 621.2 | 114.0 | 1.8 | 323.8 | 11.4 | 32.0 | 85.8 |
| *caliginosus* | Queen | 5 | 4.58 | 38.00 | 22.02 | 8.82 | 25.02 | 17.18 | 16.82 | 12.82 | 279.04 | 1029.6 | 194.0 | 2.4 | 543.4 | 16.4 | 26.8 | 86.4 |
| *caliginosus* | Male | 5 | 3.41 | 37.96 | 21.94 | 9.06 | 25.10 | 17.48 | 17.16 | 13.28 | 334.18 | 816.4 | 170.4 | 3.0 | 449.0 | 15.2 | 18.4 | 88.6 |
| *centralis* | Worker | 5 | 3.08 | 38.69 | 34.48 | -1.26 | 24.28 | 13.50 | 9.76 | 4.76 | 654.64 | 632.8 | 103.4 | 15.8 | 279.2 | 56.8 | 61.2 | 54.4 |
| *centralis* | Queen | 5 | 4.46 | 37.23 | 28.80 | -2.90 | 20.00 | 11.20 | 11.20 | 3.30 | 580.40 | 449.0 | 79.0 | 12.0 | 203.0 | 42.0 | 52.0 | 59.0 |
| *centralis* | Male | 5 | 3.04 | 37.07 | 28.40 | -2.64 | 19.94 | 11.32 | 11.26 | 3.46 | 576.26 | 524.2 | 92.6 | 12.0 | 241.4 | 42.4 | 53.6 | 62.0 |
| *crotchii* | Worker | 5 | 4.39 | 34.71 | 29.52 | 9.90 | 32.12 | 23.10 | 22.78 | 15.68 | 555.46 | 385.0 | 74.4 | 0.4 | 211.2 | 4.6 | 10.4 | 85.6 |
| *crotchii* | Queen | 5 | 6.45 | 34.43 | 24.30 | 10.98 | 28.10 | 20.52 | 19.50 | 15.24 | 395.30 | 433.8 | 84.0 | 1.2 | 240.6 | 13.6 | 22.0 | 87.6 |
| *crotchii* | Male | 5 | 4.71 | 35.46 | 31.78 | 11.02 | 34.80 | 24.88 | 24.70 | 17.16 | 598.86 | 251.0 | 47.4 | 0.4 | 132.8 | 3.2 | 4.0 | 81.6 |
| *dahlbomii* | Worker | 6 | 4.73 | 41.10 | 23.18 | 5.42 | 23.42 | 15.18 | 15.18 | 9.98 | 403.97 | 993.7 | 175.5 | 22.0 | 493.8 | 91.5 | 91.5 | 63.3 |
| *dahlbomii* | Queen | 8 | 8.02 | 38.15 | 21.89 | 6.86 | 23.66 | 15.79 | 15.65 | 11.05 | 364.11 | 1232.6 | 226.0 | 32.8 | 580.5 | 122.0 | 122.0 | 73.8 |
| *dahlbomii* | Male | 7 | 5.34 | 37.70 | 21.94 | 8.30 | 25.11 | 17.36 | 17.36 | 12.44 | 374.09 | 783.4 | 162.9 | 12.6 | 430.1 | 52.3 | 52.3 | 83.7 |
| *diligens* | Worker | 5 | 3.76 | 18.73 | 23.12 | 19.46 | 29.26 | 20.26 | 16.96 | 18.06 | 192.76 | 872.4 | 201.8 | 6.6 | 515.8 | 23.2 | 381.0 | 95.4 |
| *diligens* | Queen | 5 | 5.63 | 18.47 | 22.70 | 19.48 | 29.22 | 20.32 | 16.88 | 18.20 | 184.90 | 803.6 | 185.0 | 5.8 | 462.0 | 21.4 | 323.2 | 96.2 |
| *diligens* | Male | 7 | 5.14 | 18.40 | 18.32 | 22.13 | 29.78 | 22.95 | 19.18 | 20.80 | 196.27 | 1665.0 | 318.8 | 28.8 | 891.2 | 92.5 | 477.0 | 84.5 |
| *diversus* | Worker | 4 | 3.85 | 35.98 | 31.98 | 21.93 | 28.38 | 22.55 | 3.78 | 12.43 | 787.18 | 1743.5 | 230.8 | 72.5 | 632.0 | 240.3 | 622.3 | 37.8 |
| *diversus* | Queen | 2 | 4.59 | 36.84 | 32.90 | 15.10 | 20.90 | 15.10 | -6.70 | 4.10 | 856.80 | 2011.0 | 300.0 | 76.0 | 848.0 | 234.0 | 848.0 | 49.0 |
| *diversus* | Male | 8 | 4.73 | 35.95 | 33.15 | 19.53 | 25.58 | 19.61 | -1.09 | 8.84 | 838.25 | 1839.9 | 258.3 | 67.3 | 722.8 | 220.1 | 703.3 | 44.8 |
| *ephippiatus* | Worker | 5 | 3.64 | 20.00 | 22.70 | 19.34 | 28.56 | 2006 | 16.48 | 17.68 | 210.48 | 860.4 | 188.4 | 5.6 | 509.0 | 25.8 | 385.0 | 90.8 |
| *ephippiatus* | Queen | 6 | 5.16 | 18.96 | 21.83 | 20.18 | 29.48 | 21.07 | 17.65 | 19.02 | 182.30 | 1090.2 | 229.0 | 9.8 | 631.0 | 37.0 | 377.2 | 94.2 |
| *ephippiatus* | Male | 6 | 3.39 | 17.87 | 22.12 | 20.26 | 29.90 | 21.26 | 17.08 | 19.18 | 184.06 | 1159.8 | 242.2 | 15.6 | 611.0 | 55.4 | 328.6 | 87.4 |
| *fervidus* | Worker | 4 | 3.79 | 37.18 | 29.85 | -2.00 | 21.80 | 12.70 | 12.70 | 4.60 | 595.85 | 468.5 | 81.0 | 11.5 | 213.5 | 41.0 | 41.0 | 60.5 |
| *fervidus* | Queen | 5 | 4.62 | 38.39 | 35.70 | 8.92 | 27.78 | 17.86 | 12.00 | 8.14 | 737.78 | 502.6 | 69.2 | 18.4 | 180.2 | 64.6 | 115.4 | 41.6 |
| *fervidus* | Male | 2 | 3.73 | 37.99 | 35.20 | -1.60 | 26.20 | 15.50 | 15.50 | 6.65 | 668.25 | 316.5 | 51.5 | 11.5 | 137.0 | 39.0 | 39.0 | 51.0 |
| *flavifrons* | Worker | 5 | 3.08 | 41.14 | 40.74 | 12.86 | 25.40 | 14.56 | 0.52 | 3.88 | 817.14 | 486.2 | 57.8 | 25.4 | 156.4 | 92.8 | 144.2 | 24.8 |
| *flavifrons* | Queen | 5 | 4.12 | 48.03 | 34.28 | 2.78 | 23.80 | 14.84 | 8.30 | 4.80 | 777.66 | 1100.8 | 176.2 | 21.0 | 501.2 | 98.2 | 129.2 | 51.6 |
| *flavifrons* | Male | 5 | 3.13 | 45.41 | 22.48 | 5.28 | 23.10 | 15.94 | 15.76 | 10.22 | 437.44 | 1828.2 | 289.0 | 36.2 | 813.4 | 133.8 | 152.6 | 62.0 |
| *fraternus* | Worker | 4 | 4.08 | 36.34 | 36.60 | 18.80 | 29.45 | 23.85 | 6.78 | 13.33 | 815.48 | 904.3 | 111.8 | 45.5 | 303.0 | 150.8 | 293.0 | 33.3 |
| *fraternus* | Queen | 5 | 6.28 | 32.10 | 31.02 | 16.16 | 33.04 | 26.32 | 18.02 | 18.14 | 657.66 | 1271.6 | 142.4 | 65.4 | 398.0 | 235.0 | 392.0 | 21.8 |
| *fraternus* | Male | 6 | 5.20 | 32.20 | 31.03 | 15.07 | 33.07 | 26.27 | 17.50 | 18.13 | 647.13 | 1213.3 | 144.8 | 63.0 | 403.5 | 211.8 | 400.2 | 25.0 |
| *frigidus* | Worker | 3 | 2.93 | 62.00 | 48.97 | 8.87 | 20.30 | 12.57 | -8.50 | -3.83 | 1342.90 | 301.7 | 56.3 | 7.3 | 142.3 | 29.3 | 131.7 | 64.0 |
| *frigidus* | Queen | 4 | 4.23 | 51.82 | 40.83 | 11.03 | 18.93 | 13.13 | -9.25 | 2.00 | 994.80 | 354.8 | 69.0 | 12.3 | 170.5 | 40.8 | 152.0 | 60.5 |
| *frigidus* | Male | 5 | 3.14 | 61.60 | 37.10 | 3.48 | 16.90 | 10.42 | -5.26 | -1.90 | 979.10 | 408.6 | 63.8 | 15.6 | 165.4 | 54.0 | 130.6 | 42.8 |
| *funebris* | Worker | 5 | 4.08 | 3.91 | 15.60 | 14.52 | 21.42 | 1520 | 13.40 | 14.36 | 87.42 | 1586.0 | 241.6 | 14.8 | 680.4 | 68.8 | 380.4 | 63.2 |
| *funebris* | Queen | 4 | 6.28 | 12.27 | 20.60 | 7.90 | 15.75 | 8.00 | 5.05 | 6.95 | 121.55 | 887.5 | 147.5 | 10.5 | 419.5 | 47.0 | 376.5 | 68.5 |
| *funebris* | Male | 5 | 5.20 | 12.52 | 21.28 | 8.24 | 16.26 | 8.32 | 5.16 | 7.20 | 130.34 | 851.2 | 144.8 | 9.2 | 409.2 | 42.4 | 374.8 | 70.8 |
| *funeralis* | Worker | 5 | 6.19 | 0.13 | 11.64 | 10.60 | 16.26 | 10.68 | 9.84 | 10.40 | 35.80 | 1059.2 | 133.4 | 43.2 | 367.2 | 155.8 | 345.8 | 31.0 |
| *funeralis* | Queen | 7 | 8.82 | 0.53 | 13.37 | 12.93 | 19.60 | 13.17 | 12.59 | 12.94 | 31.84 | 1251.3 | 169.4 | 40.1 | 456.6 | 152.4 | 372.7 | 38.4 |
| *funeralis* | Male | 2 | 6.26 | 1.10 | 11.50 | 9.10 | 14.85 | 9.20 | 9.00 | 9.05 | 22.45 | 1026.0 | 135.0 | 41.0 | 368.5 | 146.0 | 310.5 | 32.0 |
| *griseocollis* | Worker | 5 | 3.69 | 45.57 | 46.14 | 17.12 | 27.20 | 19.38 | -6.82 | 5.40 | 1157.30 | 754.8 | 109.4 | 25.2 | 306.0 | 90.8 | 292.0 | 49.2 |
| *griseocollis* | Queen | 5 | 5.48 | 39.87 | 36.00 | 17.72 | 29.46 | 21.94 | 2.38 | 11.12 | 858.74 | 1035.2 | 106.0 | 62.8 | 761.0 | 206.0 | 291.4 | 15.2 |
| *griseocollis* | Male | 5 | 4.41 | 44.78 | 43.88 | 12.54 | 28.40 | 20.66 | 0.50 | 7.08 | 1095.12 | 343.0 | 94.6 | 39.0 | 258.4 | 122.6 | 236.0 | 39.6 |
| *hortorun* | Worker | 5 | 3.53 | 51.01 | 24.60 | 11.84 | 17.92 | 16.12 | 2.98 | 8.66 | 595.48 | 673.6 | 80.2 | 38.4 | 226.6 | 124.2 | 211.8 | 25.0 |
| *hortorun* | Queen | 6 | 4.76 | 50.26 | 23.48 | 8.98 | 22.17 | 16.42 | 4.12 | 9.38 | 553.20 | 692.0 | 75.8 | 44.5 | 210.7 | 143.8 | 183.2 | 16.0 |
| *hortorun* | Male | 2 | 3.49 | 51.15 | 25.30 | 16.65 | 12.55 | 16.65 | 2.90 | 8.75 | 630.50 | 627.0 | 73.0 | 38.0 | 202.0 | 120.0 | 202.0 | 20.0 |
| *hoshuensis* | Worker | 1 | 4.40 | 42.95 | 35.60 | 14.40 | 23.80 | 17.80 | 3.10 | 5.60 | 965.60 | 1349.0 | 151.0 | 81.0 | 420.0 | 248.0 | 403.0 | 22.0 |
| *hoshuensis* | Queen | 1 | 6.10 | 42.95 | 35.60 | 14.40 | 23.80 | 17.80 | 3.10 | 5.60 | 965.60 | 1349.0 | 151.0 | 81.0 | 420.0 | 248.0 | 403.0 | 22.0 |
| *hoshuensis* | Male | 1 | 4.50 | 42.95 | 35.60 | 14.40 | 23.80 | 17.80 | 3.10 | 5.60 | 965.60 | 1349.0 | 151.0 | 81.0 | 420.0 | 248.0 | 403.0 | 22.0 |
| *huntii* | Worker | 5 | 3.22 | 37.60 | 37.90 | 4.30 | 30.66 | 20.30 | 10.44 | 10.02 | 773.78 | 388.4 | 49.2 | 12.0 | 135.6 | 55.4 | 93.6 | 32.6 |
| *huntii* | Queen | 5 | 4.66 | 37.31 | 36.86 | 5.76 | 30.74 | 20.68 | 11.96 | 10.54 | 762.84 | 328.8 | 43.2 | 9.2 | 121.0 | 42.4 | 83.6 | 40.8 |
| *huntii* | Male | 5 | 3.13 | 35.94 | 32.86 | 11.72 | 28.74 | 18.88 | 9.64 | 10.72 | 610.26 | 491.2 | 86.8 | 7.6 | 242.4 | 36.2 | 55.0 | 59.4 |
| *hyperboreus* | Worker | 5 | 3.34 | 68.20 | 39.90 | 5.30 | 11.72 | 6.06 | -19.56 | -8.86 | 1155.36 | 274.6 | 42.6 | 12.8 | 111.4 | 41.8 | 102.6 | 57.6 |
| *hyperboreus* | Queen | 5 | 6.04 | 65.70 | 42.76 | 8.72 | 15.96 | 9.38 | -16.40 | -6.06 | 1208.24 | 341.2 | 52.4 | 14.6 | 139.0 | 49.2 | 134.4 | 53.4 |
| *hyperboreus* | Male | 3 | 3.53 | 68.55 | 44.57 | 6.20 | 13.07 | 6.87 | -21.83 | -9.60 | 1285.87 | 194.7 | 37.0 | 7.0 | 91.3 | 24.0 | 86.0 | 64.0 |
| *hypnorum* | Worker | 5 | 3.34 | 55.38 | 25.82 | 9.50 | 20.78 | 15.02 | 1.12 | 6.80 | 638.20 | 697.4 | 80.0 | 38.8 | 224.2 | 126.6 | 211.6 | 22.0 |
| *hypnorum* | Queen | 5 | 5.14 | 49.60 | 25.16 | 13.78 | 22.00 | 16.06 | 1.86 | 8.34 | 608.14 | 808.2 | 96.2 | 48.6 | 269.4 | 154.6 | 267.8 | 21.2 |
| *hypnorum* | Male | 3 | 3.53 | 49.16 | 25.73 | 10.67 | 20.43 | 14.47 | 0.47 | 6.73 | 610.83 | 967.3 | 120.3 | 53.7 | 343.0 | 174.0 | 340.3 | 25.3 |
| *hypocrita* | Worker | 3 | 5.40 | 35.96 | 33.83 | 24.10 | 30.37 | 24.17 | 4.70 | 13.27 | 850.40 | 1372.7 | 196.7 | 59.3 | 517.3 | 189.7 | 504.3 | 41.0 |
| *hypocrita* | Queen | 3 | 7.70 | 35.96 | 33.83 | 24.10 | 30.37 | 24.17 | 4.70 | 13.27 | 850.40 | 1372.7 | 196.7 | 59.3 | 517.3 | 189.7 | 504.3 | 41.0 |
| *hypocrita* | Male | 3 | 5.80 | 35.96 | 33.83 | 24.10 | 30.37 | 24.17 | 4.70 | 13.27 | 850.40 | 1372.7 | 196.7 | 59.3 | 517.3 | 189.7 | 504.3 | 41.0 |
| *ignutus* | Worker | 1 | 6.30 | 36.81 | 33.60 | 23.20 | 29.30 | 23.20 | 2.20 | 12.70 | 823.60 | 1448.0 | 199.0 | 47.0 | 592.0 | 154.0 | 592.0 | 48.0 |
| *ignutus* | Queen | 1 | 8.10 | 36.81 | 33..60 | 23.20 | 29.30 | 23.20 | 2.20 | 12.70 | 823.60 | 1448.0 | 199.0 | 47.0 | 592.0 | 154.0 | 592.0 | 48.0 |
| *ignutus* | Male | 1 | 6.40 | 36.81 | 33.60 | 23.20 | 29.30 | 23.20 | 2.20 | 12.70 | 823.60 | 1448.0 | 199.0 | 47.0 | 592.0 | 154.0 | 592.0 | 48.0 |
| *impatiens* | Worker | 5 | 3.38 | 31.57 | 28.56 | 23.18 | 32.36 | 26.78 | 15.90 | 18.86 | 645.90 | 1442.6 | 162.4 | 77.2 | 459.2 | 277.4 | 447.4 | 21.0 |
| *impatiens* | Queen | 5 | 5.21 | 37.68 | 34.76 | 18.32 | 30.08 | 23.22 | 6.34 | 12.96 | 822.66 | 1203.0 | 129.2 | 72.2 | 362.4 | 238.6 | 350.0 | 18.4 |
| *impatiens* | Male | 5 | 3.42 | 41.43 | 37.44 | 15.70 | 27.80 | 20.26 | -2.66 | 9.02 | 904.56 | 1014.8 | 102.2 | 65.4 | 295.8 | 211.2 | 285.4 | 14.4 |
| *jonellus* | Worker | 5 | 3.16 | 54.71 | 27.72 | 8.44 | 20.92 | 15.06 | 4.30 | 6.36 | 684.06 | 741.2 | 84.2 | 45.4 | 178.4 | 144.8 | 207.6 | 23.4 |
| *jonellus* | Queen | 5 | 4.76 | 58.12 | 22.40 | 6.54 | 18.38 | 13.34 | 6.32 | 6.02 | 551.06 | 941.2 | 103.0 | 58.4 | 249.0 | 185.2 | 242.8 | 19.0 |
| *jonellus* | Male | 5 | 2.81 | 58.08 | 24.76 | 5.02 | 17.18 | 12.24 | 2.38 | 3.74 | 648.14 | 718.2 | 78.6 | 45.8 | 163.8 | 145.2 | 184.2 | 27.2 |
| *lapidarium* | Worker | 8 | 3.16 | 51.62 | 21.41 | 8.49 | 21.35 | 16.16 | 5.44 | 9.75 | 501.04 | 679.0 | 74.1 | 41.3 | 211.8 | 133.3 | 194.4 | 17.3 |
| *lapidarium* | Queen | 6 | 5.19 | 49.24 | 26.78 | 12.58 | 22.45 | 16.15 | 1.57 | 8.18 | 634.70 | 959.8 | 117.7 | 54.7 | 333.3 | 176.8 | 321.0 | 26.0 |
| *lapidarium* | Male | 2 | 3.03 | 47.86 | 29.75 | 17.05 | 23.90 | 17.05 | 0.45 | 8.25 | 701.25 | 1269.0 | 163.5 | 73.0 | 467.5 | 236.5 | 467.5 | 30.0 |
| *lapponicus* | Worker | 1 | 2.87 | 53.90 | 41.90 | 16.70 | 23.97 | 16.70 | -9.80 | 2.90 | 1112.97 | 497.7 | 79.0 | 19.3 | 206.7 | 65.3 | 206.7 | 48.3 |
| *lapponicus* | Queen | 1 | 3.85 | 53.90 | 41.90 | 16.70 | 23.97 | 16.70 | -9.80 | 2.90 | 1112.97 | 497.7 | 79.0 | 19.3 | 206.7 | 65.3 | 206.7 | 48.3 |
| *lapponicus* | Male | 3 | 3.06 | 53.90 | 41.90 | 16.70 | 23.98 | 16.70 | -980 | 2.90 | 1112.98 | 497.8 | 79.0 | 19.2 | 206.8 | 65.2 | 206.8 | 48.2 |
| *lucorum* | Worker | 4 | 3.75 | 65.42 | 22.78 | 12.35 | 26.18 | 21.03 | 14.10 | 13.83 | 607.33 | 645.5 | 106.3 | 15.0 | 297.3 | 49.5 | 95.0 | 58.0 |
| *lucorum* | Queen | 6 | 4.42 | 53.87 | 29.75 | 7.23 | 18.37 | 12.63 | -6.47 | 2.32 | 805.20 | 510.5 | 61.2 | 28.7 | 171.5 | 95.3 | 149.2 | 32.8 |
| *lucorum* | Male | 5 | 4.15 | 44.63 | 35.98 | 12.96 | 19.56 | 13.38 | -11.44 | 3.8 | 1017.80 | 461.8 | 60.4 | 24.8 | 162.2 | 80.6 | 159.8 | 36.2 |
| *medius* | Worker | 5 | 3.64 | 19.29 | 19.12 | 23.12 | 30.64 | 23.82 | 18.82 | 21.42 | 227.94 | 2125.8 | 411.8 | 41.4 | 1104.8 | 135.2 | 578.6 | 77.6 |
| *medius* | Queen | 1 | 5.66 | 16.23 | 17.80 | 24.50 | 32.50 | 25.00 | 32.50 | 23.60 | 133.90 | 2766.0 | 463.0 | 48.0 | 1334.0 | 168.0 | 672.0 | 73.0 |
| *medius* | Male | 4 | 3.24 | 20.52 | 18.05 | 26.05 | 32.85 | 26.63 | 22.15 | 24.38 | 229.18 | 1478.5 | 283.3 | 35.8 | 712.5 | 117.8 | 550.3 | 69.8 |
| *melaleucos* | Worker | 6 | 6.87 | 6.40 | 14.77 | 18.07 | 23.95 | 18.62 | 14.78 | 16.40 | 182.40 | 1564.3 | 241.3 | 36.8 | 627.7 | 145.7 | 379.7 | 64.7 |
| *melaleucos* | Queen | 4 | 10.39 | 0.72 | 12.63 | 11.73 | 17.93 | 11.88 | 11.35 | 11.65 | 23.88 | 1097.8 | 160.5 | 27.5 | 439.8 | 118.3 | 403.0 | 45.3 |
| *melaleucos* | Male | 1 | 8.15 | 8.58 | 12.30 | 25.40 | 32.30 | 26.70 | 25.80 | 25.80 | 64.90 | 3402.0 | 631.0 | 33.0 | 1538.0 | 184.0 | 572.0 | 65.0 |
| *melanopygus* | Worker | 5 | 3.15 | 34.14 | 27.00 | 3.74 | 24.56 | 16.34 | 14.50 | 9.30 | 515.84 | 649.6 | 113.8 | 4.4 | 327.4 | 38.2 | 67.2 | 77.4 |
| *melanopygus* | Queen | 5 | 4.82 | 34.07 | 18.32 | 14.30 | 27.12 | 21.60 | 20.90 | 17.50 | 292.94 | 391.6 | 85.2 | 0.0 | 234.8 | 5.0 | 11.0 | 95.8 |
| *melanopygus* | Male | 5 | 2.96 | 32.61 | 24.24 | 8.88 | 26.20 | 18.96 | 17.44 | 13.44 | 406.30 | 445.4 | 87.4 | 1.6 | 242.2 | 17.6 | 26.2 | 85.2 |
| *mesomelas* | Worker | 5 | 3.88 | 47.02 | 25.86 | 14.06 | 21.18 | 1546 | -0.22 | 7.26 | 651.20 | 1068.6 | 126.6 | 65.8 | 359.4 | 204.8 | 341.2 | 25.0 |
| *mesomelas* | Queen | 2 | 4.95 | 44.69 | 24.30 | 11.40 | 20.45 | 14.90 | 0.05 | 7.05 | 608.30 | 1131.5 | 130.5 | 71.5 | 369.5 | 222.5 | 324.0 | 21.5 |
| *mesomelas* | Male | 1 | 4.10 | 46.81 | 23.60 | 12.80 | 18.00 | 12.80 | -1.50 | 5.30 | 588.40 | 1426.0 | 160.0 | 93.0 | 458.0 | 288.0 | 458.0 | 19.0 |
| *mexicanus* | Worker | 5 | 3.64 | 16.09 | 18.24 | 21.48 | 29.72 | 22.34 | 19.68 | 20.62 | 148.76 | 1563.8 | 285.0 | 24.2 | 762.0 | 85.8 | 315.8 | 84.6 |
| *mexicanus* | Queen | 5 | 6.05 | 15.74 | 16.42 | 24.12 | 31.78 | 24.82 | 22.40 | 23.64 | 100.58 | 3183.0 | 551.0 | 26.6 | 1476.2 | 101.2 | 678.4 | 80.2 |
| *mexicanus* | Male | 2 | 3.12 | 14.10 | 15.75 | 21.45 | 29.20 | 22.20 | 20.75 | 21.15 | 88.20 | 1602.0 | 318.5 | 3.5 | 831.0 | 16.5 | 335.5 | 92.5 |
| *mixtus* | Worker | 5 | 3.10 | 44.56 | 28.20 | 7.8 | 23.96 | 15.56 | 15.46 | 7.66 | 599.92 | 917.0 | 145.2 | 22.0 | 409.8 | 82.6 | 88.0 | 56.8 |
| *mixtus* | Queen | 5 | 4.27 | 37.98 | 31.48 | 8.4 | 26.18 | 16.26 | 16.18 | 7.96 | 621.92 | 889.2 | 159.0 | 7.8 | 435.0 | 34.2 | 42.6 | 75.0 |
| *mixtus* | Male | 5 | 2.95 | 51.54 | 35.54 | 5.92 | 21.40 | 13.58 | 2.16 | 2.06 | 891.10 | 904.6 | 159.0 | 11.2 | 443.0 | 51.0 | 92.0 | 66.6 |
| *morio* | Worker | 8 | 4.07 | 7.84 | 18.24 | 18.76 | 27.94 | 21.16 | 11.18 | 11.58 | 221.56 | 979.0 | 148.8 | 20.2 | 407.8 | 79.2 | 161.4 | 54.0 |
| *morio* | Queen | 5 | 4.64 | 32.37 | 24.03 | 17.37 | 28.47 | 21.07 | 8.80 | 9.50 | 416.13 | 981.7 | 166.3 | 20.3 | 452.0 | 77.0 | 188.8 | 65.3 |
| *morio* | Male | 3 | 4.27 | 15.62 | 21.23 | 24.03 | 31.43 | 24.47 | 16.53 | 20.57 | 310.10 | 1075.0 | 148.7 | 26.3 | 405.3 | 95.7 | 354.0 | 48.3 |
| *morrisoni* | Worker | 17 | 4.20 | 37.00 | 38.04 | 7.36 | 32.22 | 21.75 | 15.54 | 11.56 | 771.88 | 319.3 | 47.9 | 8.4 | 126.9 | 37.0 | 65.6 | 43.6 |
| *morrisoni* | Queen | 15 | 6.55 | 37.01 | 36.87 | 6.79 | 29.32 | 18.59 | 13.89 | 9.01 | 718.18 | 402.0 | 63.1 | 11.1 | 166.2 | 45.7 | 91.8 | 43.9 |
| *morrisoni* | Male | 11 | 4.66 | 37.28 | 36.53 | 10.24 | 27.98 | 17.68 | 9.00 | 8.21 | 714.28 | 363.1 | 60.5 | 10.9 | 155.7 | 39.0 | 91.1 | 52.7 |
| *nevadensis* | Worker | 5 | 5.12 | 37.92 | 40.54 | 6.38 | 25.66 | 15.18 | 9.24 | 4.72 | 825.14 | 451.2 | 64.0 | 23.6 | 170.0 | 81.0 | 147.4 | 34.8 |
| *nevadensis* | Queen | 4 | 6.05 | 42.83 | 31.18 | 5.58 | 29.88 | 20.60 | 18.75 | 12.45 | 625.73 | 536.3 | 87.5 | 9.8 | 248.8 | 44.5 | 49.0 | 52.8 |
| *nevadensis* | Male | 5 | 5.20 | 40.48 | 35.92 | 2.02 | 22.56 | 12.64 | 9.04 | 3.00 | 740.38 | 506.6 | 62.8 | 24.8 | 171.0 | 87.8 | 103.8 | 31.4 |
| *ocidentalis* | Worker | 5 | 3.54 | 39.67 | 35.02 | 5.76 | 30.68 | 19.98 | 15.16 | 10.52 | 707.48 | 642.6 | 116.2 | 7.2 | 314.6 | 28.4 | 46.0 | 66.4 |
| *ocidentalis* | Queen | 5 | 4.99 | 37.64 | 33.40 | 12.32 | 29.98 | 20.08 | 11.48 | 11.62 | 636.56 | 554.2 | 79.4 | 20.6 | 201.8 | 70.4 | 116.0 | 53.2 |
| *ocidentalis* | Male | 5 | 3.56 | 38.28 | 33.92 | 5.16 | 23.26 | 13.72 | 5.40 | 4.68 | 678.90 | 727.8 | 127.6 | 10.6 | 345.6 | 42.6 | 113.8 | 66.4 |
| *pennsylvanicus* | Worker | 5 | 3.97 | 34.41 | 32.30 | 24.18 | 32.06 | 25.92 | 10.48 | 16.46 | 763.78 | 1260.6 | 152.6 | 65.8 | 404.4 | 229.4 | 369.4 | 25.2 |
| *pennsylvanicus* | Queen | 5 | 5.43 | 33.60 | 32.32 | 25.10 | 32.62 | 26.08 | 10.06 | 17.20 | 721.94 | 1171.6 | 170.0 | 44.8 | 456.6 | 153.8 | 446.6 | 41.0 |
| *pennsylvanicus* | Male | 5 | 4.28 | 33.90 | 32.78 | 14.58 | 31.96 | 24.68 | 13.36 | 15.76 | 709.10 | 1115.0 | 122.6 | 60.6 | 347.2 | 206.4 | 334.6 | 26.4 |
| *perplexus* | Worker | 5 | 3.18 | 40.80 | 36.50 | 15.54 | 26.92 | 19.72 | -2.28 | 8.70 | 876.94 | 1173.2 | 110.6 | 81.0 | 319.2 | 258.6 | 308.8 | 9.0 |
| *perplexus* | Queen | 4 | 4.98 | 38.70 | 34.33 | 8.45 | 27.50 | 20.38 | 6.25 | 10.35 | 801.30 | 1530.5 | 150.3 | 109.3 | 411.5 | 348.8 | 383.0 | 8.8 |
| *perplexus* | Male | 5 | 3.11 | 41.95 | 37.84 | 9.76 | 27.78 | 20.10 | 1.48 | 8.72 | 904.22 | 1133.0 | 108.8 | 79.4 | 308.4 | 251.8 | 291.6 | 9.2 |
| *polaris* | Worker | 5 | 4.07 | 57.81 | 43.58 | 12.66 | 20.16 | 12.96 | -10.74 | -0.98 | 1144.50 | 468.8 | 73.4 | 19.6 | 198.6 | 64.4 | 194.8 | 45.8 |
| *polaris* | Queen | 3 | 5.06 | 54.93 | 34.70 | 11.17 | 18.43 | 11.67 | -7.73 | 1.57 | 827.97 | 586.7 | 98.7 | 23.0 | 271.3 | 77.0 | 265.0 | 55.3 |
| *polaris* | Male | 2 | 2.71 | 55.49 | 30.55 | 9.95 | 16.50 | 10.70 | -6.75 | 1.55 | 738.70 | 591.5 | 94.0 | 25.0 | 260.0 | 81.0 | 250.5 | 50.5 |
| *pratorum* | Worker | 5 | 3.08 | 52.25 | 20.54 | 4.06 | 20.30 | 15.12 | 5.44 | 9.10 | 472.42 | 685.4 | 70.6 | 44.8 | 197.6 | 145.4 | 171.0 | 12.6 |
| *pratorum* | Queen | 5 | 4.38 | 51.69 | 21.38 | 6.54 | 20.54 | 15.10 | 4.36 | 8.78 | 496.24 | 707.0 | 75.8 | 45.6 | 211.2 | 147.6 | 181.6 | 14.6 |
| *pratorum* | Male | 5 | 3.05 | 52.00 | 20.46 | 4.14 | 20.30 | 14.98 | 5.30 | 9.00 | 465.52 | 719.4 | 74.0 | 47.2 | 208.2 | 153.8 | 177.0 | 12.2 |
| *pseudobaicalensis* | Worker | 1 | 4.60 | 44.01 | 32.90 | 8.40 | 15.60 | 10.80 | -11.80 | -0.50 | 983.20 | 1444.0 | 196.0 | 60.0 | 517.0 | 207.0 | 510.0 | 33.0 |
| *pseudobaicalensis* | Queen | 1 | 6.50 | 44.01 | 32.90 | 8.40 | 15.60 | 10.80 | -11.80 | -0.50 | 983.20 | 1444.0 | 196.0 | 60.0 | 517.0 | 207.0 | 510.0 | 33.0 |
| *pseudobaicalensis* | Male | 1 | 4.80 | 44.01 | 32.90 | 8.40 | 15.60 | 10.80 | -11.80 | -0.50 | 983.20 | 1444.0 | 196.0 | 60.0 | 517.0 | 207.0 | 510.0 | 33.0 |
| *pullatus* | Worker | 5 | 4.49 | 11.34 | 12.54 | 25.62 | 32.14 | 26.60 | 25.98 | 25.40 | 87.84 | 3780.0 | 458.6 | 146.0 | 1263.8 | 524.8 | 738.0 | 34.4 |
| *pullatus* | Queen | 3 | 7.21 | 10.44 | 12.17 | 24.00 | 30.03 | 24.73 | 23.87 | 23.77 | 76.73 | 3007.7 | 356.7 | 117.0 | 974.7 | 424.3 | 570.0 | 36.3 |
| *pullatus* | Male | 7 | 4.28 | 8.96 | 13.11 | 20.34 | 27.43 | 21.34 | 20.41 | 20.51 | 62.54 | 2873.0 | 533.3 | 21.7 | 1289.3 | 108.1 | 552.3 | 67.7 |
| *robustus* | Worker | 4 | 6.15 | 1.49 | 10.00 | 4.30 | 9.30 | 4.30 | 3.50 | 4.10 | 33.60 | 651.0 | 93.0 | 20.0 | 270.0 | 74.0 | 222.0 | 44.0 |
| *robustus* | Queen | 3 | 9.16 | 0.75 | 10.10 | 6.00 | 11.00 | 6.00 | 5.17 | 5.77 | 36.03 | 808.0 | 105.7 | 32.7 | 298.3 | 115.3 | 266.3 | 37.3 |
| *robustus* | Male | 2 | 6.86 | 2.23 | 11.00 | 25.90 | 30.10 | 26.10 | 23.60 | 24.10 | 147.80 | 218.0 | 85.0 | 1.0 | 181.0 | 3.0 | 178.0 | 146.0 |
| *rofocinatus* | Worker | 5 | 3.13 | 39.61 | 37.00 | 5.18 | 24.66 | 14.88 | 6.96 | 4.86 | 781.76 | 516.0 | 81.6 | 14.2 | 218.2 | 52.4 | 111.8 | 51.0 |
| *rofocinatus* | Queen | 5 | 4.60 | 41.17 | 36.10 | 3.74 | 25.96 | 15.54 | 10.08 | 5.96 | 728.76 | 470.6 | 72.4 | 17.6 | 190.2 | 60.4 | 90.8 | 44.6 |
| *rofocinatus* | Male | 6 | 3.64 | 39.63 | 34.78 | 1.87 | 26.38 | 15.67 | 14.47 | 6.80 | 675.97 | 443.8 | 68.2 | 14.3 | 184.5 | 54.5 | 59.3 | 47.8 |
| *rubicundus* | Worker | 5 | 3.98 | 0.54 | 13.26 | 10.88 | 16.86 | 11.10 | 9.82 | 10.56 | 61.36 | 1177.0 | 164.0 | 29.2 | 461.8 | 115.6 | 381.6 | 46.4 |
| *rubicundus* | Queen | 2 | 6.11 | 16.99 | 16.40 | 23.30 | 29.45 | 23.35 | 20.20 | 21.80 | 166.55 | 1222.5 | 198.5 | 34.5 | 540.0 | 118.5 | 496.5 | 52.5 |
| *rubicundus* | Male | 4 | 4.41 | 2.96 | 12.25 | 7.20 | 12.80 | 7.35 | 6.15 | 6.85 | 57.10 | 970.0 | 132.5 | 29.5 | 368.0 | 107.0 | 288.0 | 42.5 |
| *schrencki* | Worker | 1 | 4.80 | 43.05 | 32.20 | 14.70 | 21.50 | 16.60 | -5.00 | 5.90 | 840.20 | 189.0 | 165.0 | 41.0 | 409.0 | 147.0 | 392.0 | 37.0 |
| *schrencki* | Queen | 1 | 7.90 | 43.05 | 32.20 | 14.70 | 21.50 | 16.60 | -5.00 | 5.90 | 840.20 | 189.0 | 165.0 | 41.0 | 409.0 | 147.0 | 392.0 | 37.0 |
| *schrencki* | Male | 1 | 4.50 | 43.05 | 32.20 | 14.70 | 21.50 | 16.60 | -5.00 | 5.90 | 840.20 | 189.0 | 165.0 | 41.0 | 409.0 | 147.0 | 392.0 | 37.0 |
| *sichelii* | Worker | 5 | 3.16 | 46.78 | 27.92 | 16.38 | 22.82 | 16.76 | -0.34 | 7.80 | 706.84 | 997.4 | 118.6 | 63.0 | 332.2 | 199.2 | 331.2 | 21.8 |
| *sichelii* | Queen | 4 | 5.05 | 46.94 | 24.80 | 15.00 | 20.70 | 15.00 | 0.30 | 7.20 | 612.70 | 1192.0 | 142.0 | 76.0 | 398.0 | 239.0 | 398.0 | 22.0 |
| *sichelii* | Male | 5 | 3.86 | 31.23 | 32.24 | 22.06 | 32.82 | 26.68 | 11.10 | 16.84 | 804.36 | 1498.2 | 264.2 | 40.6 | 735.4 | 148.0 | 484.8 | 54.6 |
| *sitkensis* | Worker | 15 | 3.17 | 44.91 | 20.99 | 5.95 | 22.74 | 16.01 | 15.81 | 10.81 | 410.07 | 2601.1 | 444.3 | 44.3 | 1200.5 | 166.1 | 197.7 | 68.9 |
| *sitkensis* | Queen | 9 | 4.38 | 40.68 | 19.98 | 7.97 | 22.97 | 16.17 | 15.94 | 11.80 | 339.04 | 1356.0 | 237.8 | 9.9 | 667.3 | 47.3 | 62.4 | 78.3 |
| *sitkensis* | Male | 15 | 3.15 | 42.56 | 18.09 | 7.63 | 21.10 | 15.25 | 14.95 | 11.13 | 317.07 | 1765.0 | 306.5 | 21.0 | 837.9 | 89.1 | 107.4 | 72.7 |
| *sonorus* | Worker | 5 | 3.94 | 34.18 | 17.66 | 14.16 | 26.12 | 20.74 | 18.84 | 17.12 | 266.54 | 327.6 | 73.4 | 0.0 | 197.4 | 3.4 | 7.6 | 99.2 |
| *sonorus* | Queen | 5 | 6.08 | 33.76 | 18.68 | 13.82 | 26.42 | 20.94 | 19.62 | 17.02 | 290.04 | 314.8 | 68.0 | 0.0 | 186.8 | 4.0 | 8.8 | 95.0 |
| *sonorus* | Male | 5 | 4.16 | 34.09 | 18.04 | 13.70 | 26.00 | 20.52 | 19.82 | 16.74 | 273.90 | 362.8 | 81.6 | 0.0 | 217.6 | 3.4 | 7.4 | 98.6 |
| *steindachneri* | Worker | 12 | 3.68 | 21.14 | 22.24 | 21.18 | 30.60 | 21.93 | 18.05 | 19.31 | 220.08 | 1123.9 | 274.3 | 5.3 | 736.6 | 26.0 | 489.2 | 105.3 |
| *steindachneri* | Queen | 12 | 5.67 | 21.23 | 24.09 | 22.36 | 32.13 | 23.08 | 19.41 | 20.31 | 238.58 | 922.6 | 230.8 | 4.6 | 607.8 | 19.3 | 418.1 | 108.3 |
| *steindachneri* | Male | 10 | 3.95 | 26.31 | 29.48 | 27.62 | 37.77 | 28.16 | 22.56 | 29.22 | 421.69 | 647.6 | 178.5 | 3.3 | 451.0 | 17.0 | 377.3 | 111.0 |
| *subterraneus* | Worker | 1 | 3.77 | 53.49 | 19.40 | 4.40 | 19.90 | 15.00 | 13.50 | 9.00 | 451.70 | 843.0 | 85.0 | 59.0 | 146.0 | 185.0 | 197.0 | 12.0 |
| *subterraneus* | Queen | 4 | 5.64 | 51.39 | 24.60 | 13.28 | 21.68 | 15.93 | 3.75 | 8.25 | 604.28 | 670.0 | 79.8 | 40.5 | 199.0 | 127.0 | 211.8 | 24.0 |
| *subterraneus* | Male | 5 | 3.74 | 53.94 | 25.04 | 10.74 | 20.90 | 15.34 | 4.66 | 7.28 | 62244 | 722.0 | 84.0 | 44.2 | 196.8 | 138.4 | 215.4 | 22.8 |
| *sylvarum* | Worker | 5 | 3.18 | 54.50 | 28.16 | 12.78 | 21.12 | 15.10 | 0.46 | 6.20 | 687.28 | 816.2 | 95.8 | 44.2 | 273.6 | 144.4 | 262.8 | 24.4 |
| *sylvarum* | Queen | 5 | 4.52 | 52.91 | 28.08 | 15.56 | 22.02 | 15.86 | -0.72 | 6.84 | 706.30 | 725.2 | 91.8 | 39.0 | 257.2 | 128.4 | 255.8 | 27.8 |
| *sylvarum* | Male | 4 | 3.33 | 54.33 | 25.10 | 12.50 | 21.05 | 16.05 | 1.75 | 8.05 | 624.80 | 646.5 | 74.0 | 36.5 | 210.0 | 120.0 | 190.0 | 22.5 |
| *sylvicola* | Worker | 5 | 3.18 | 39.15 | 37.82 | -5.90 | 19.48 | 10.00 | 2.64 | -0.34 | 795.32 | 647.0 | 69.2 | 34.4 | 188.4 | 129.4 | 151.0 | 17.4 |
| *sylvicola* | Queen | 5 | 4.13 | 51.08 | 33.90 | 5.46 | 19.18 | 11.32 | 0.54 | 0.74 | 814.26 | 772.2 | 128.2 | 19.0 | 350.6 | 74.6 | 119.0 | 52.4 |
| *sylvicola* | Male | 5 | 3.12 | 38.15 | 26.90 | -4.2 | 19.74 | 11.54 | 11.30 | 4.42 | 527.70 | 822.4 | 143.6 | 14.8 | 387.8 | 51.2 | 57.8 | 70.2 |
| *ternarius* | Worker | 5 | 3.10 | 43.41 | 40.90 | 2.12 | 27.32 | 18.96 | -4.66 | 6.92 | 960.50 | 1070.2 | 106.2 | 75.6 | 294.0 | 234.2 | 277.6 | 9.4 |
| *ternarius* | Queen | 2 | 4.72 | 45.99 | 44.10 | 16.10 | 25.40 | 17.30 | -11.20 | 3.90 | 1100.60 | 809.0 | 110.0 | 24.0 | 305.0 | 94.0 | 301.0 | 42.0 |
| *ternarius* | Male | 5 | 3.07 | 43.50 | 40.70 | 5.02 | 27.52 | 19.38 | -0.94 | 7.26 | 971.60 | 1007.2 | 106.2 | 64.8 | 295.2 | 205.0 | 269.4 | 16.0 |
| *terrestris* | Worker | 10 | 4.44 | 30.45 | 24.54 | 11.83 | 22.97 | 16.77 | 8.00 | 9.60 | 559.96 | 874.9 | 100.6 | 46.5 | 280.9 | 162.1 | 209.3 | 22.7 |
| *terrestris* | Queen | 9 | 5.70 | 8.97 | 22.27 | 9.73 | 24.54 | 18.54 | 15.89 | 12.44 | 473.89 | 979.3 | 110.7 | 50.7 | 297.3 | 181.6 | 198.1 | 26.9 |
| *terrestris* | Male | 4 | 4.41 | 48.71 | 26.68 | 15.38 | 24.28 | 17.93 | 5.20 | 9.68 | 646.30 | 581.3 | 76.5 | 23.5 | 217.0 | 86.8 | 180.3 | 36.3 |
| *terricoloa* | Worker | 5 | 3.43 | 47.43 | 4838 | 18.14 | 26.08 | 18.14 | -13.42 | 3.46 | 1219.28 | 668.0 | 105.8 | 13.8 | 296.0 | 56.4 | 296.0 | 56.6 |
| *terricoloa* | Queen | 7 | 4.83 | 43.70 | 40.49 | 12.87 | 26.36 | 18.49 | -2.43 | 6.36 | 974.49 | 1089.7 | 111.0 | 72.7 | 313.7 | 231.1 | 292.6 | 16.0 |
| *terricoloa* | Male | 5 | 3.72 | 46.45 | 44.76 | 16.72 | 2564 | 17.78 | -10.70 | 4.20 | 1116.76 | 767.4 | 107.0 | 27.0 | 292.8 | 100.4 | 285.6 | 42.0 |
| *transversalis* | Worker | 7 | 5.18 | 5.70 | 13.27 | 23.57 | 29.79 | 24.00 | 22.61 | 23.34 | 60.09 | 2062.1 | 275.7 | 84.0 | 771.1 | 282.4 | 560.6 | 38.4 |
| *transversalis* | Queen | 5 | 9.23 | 1.92 | 10.68 | 25.00 | 30.74 | 25.80 | 24.84 | 25.18 | 51.86 | 2753.2 | 355.6 | 137.0 | 970.8 | 461.0 | 561.8 | 33.8 |
| *transversalis* | Male | 2 | 5.38 | 3.27 | 10.40 | 25.05 | 30.85 | 25.90 | 24.90 | 25.30 | 49.35 | 2822.0 | 334.0 | 159.0 | 915.5 | 561.5 | 627.5 | 25.0 |
| *vagans* | Worker | 5 | 3.22 | 44.77 | 44.08 | 19.32 | 27.12 | 19.32 | -8.74 | 5.94 | 1093.10 | 834.0 | 104.4 | 38.4 | 295.2 | 130.4 | 295.2 | 38.6 |
| *vagans* | Queen | 5 | 4.39 | 44.96 | 43.60 | 16.72 | 2620 | 18.30 | -9.04 | 5.18 | 1075.36 | 906.0 | 108.0 | 43.4 | 307.0 | 166.6 | 304.8 | 34.2 |
| *vagans* | Male | 5 | 2.94 | 44.59 | 42.28 | 15.92 | 27.28 | 19.28 | -6.98 | 6.44 | 1040.00 | 823.2 | 100.2 | 39.2 | 279.4 | 131.0 | 275.4 | 31.8 |
| *vandykei* | Worker | 5 | 3.00 | 36.05 | 32.90 | 4.70 | 29.96 | 19.62 | 18.52 | 11.38 | 618.86 | 617.6 | 108.8 | 2.8 | 307.8 | 26.4 | 31.6 | 76.0 |
| *vandykei* | Queen | 5 | 3.94 | 38.66 | 24.44 | 6.92 | 25.42 | 17.36 | 17.02 | 11.52 | 433.46 | 1298.4 | 227.4 | 4.8 | 653.8 | 37.8 | 51.0 | 78.8 |
| *vandykei* | Male | 5 | 2.98 | 35.17 | 32.84 | 4.40 | 29.60 | 19.58 | 19.52 | 11.14 | 629.86 | 657.6 | 119.4 | 3.6 | 337.4 | 26.0 | 38.4 | 78.0 |
| *veteranus* | Worker | 5 | 3.24 | 50.90 | 26.04 | 16.28 | 22.24 | 16.28 | 0.68 | 8.12 | 648.40 | 608.6 | 76.2 | 35.2 | 209.6 | 109.8 | 209.6 | 25.8 |
| *veteranus* | Queen | 5 | 4.96 | 51.58 | 26.38 | 14.76 | 21.80 | 16.14 | 0.70 | 7.86 | 656.92 | 634.0 | 80.2 | 34.6 | 225.2 | 108.4 | 217.0 | 29.4 |
| *veteranus* | Male | 4 | 3.18 | 50.17 | 26.50 | 15.68 | 21.48 | 15.68 | -0.38 | 7.38 | 667.93 | 802.8 | 97.3 | 48.3 | 270.8 | 150.8 | 270.8 | 25.3 |
| *volucelloides* | Worker | 7 | 4.16 | 9.02 | 16.29 | 22.79 | 30.46 | 23.50 | 21.80 | 22.49 | 83.07 | 2178.0 | 328.9 | 52.6 | 891.3 | 190.3 | 482.0 | 58.6 |
| *volucelloides* | Queen | 6 | 6.38 | 10.20 | 12.65 | 18.28 | 25.10 | 19.37 | 18.77 | 18.43 | 70.38 | 3185.2 | 471.8 | 69.2 | 1249.0 | 237.5 | 530.7 | 50.5 |
| *volucelloides* | Male | 5 | 4.09 | 7.85 | 12.64 | 19.54 | 26.40 | 20.56 | 20.18 | 19.72 | 64.28 | 3111.2 | 429.0 | 93.0 | 1151.2 | 310.0 | 468.4 | 43.2 |
| *vosnesenskii* | Worker | 5 | 3.41 | 43.27 | 27.92 | 3.38 | 25.66 | 17.38 | 16.96 | 9.76 | 572.36 | 1222.4 | 198.0 | 19.4 | 571.6 | 88.6 | 95.6 | 61.2 |
| *vosnesenskii* | Queen | 5 | 5.30 | 37.81 | 25.14 | 6.26 | 25.66 | 17.74 | 17.16 | 11.34 | 473.50 | 1011.4 | 183.8 | 5.8 | 512.8 | 32.0 | 41.6 | 81.6 |
| *vosnesenskii* | Male | 5 | 2.98 | 35.66 | 29.06 | 28.80 | 26.52 | 17.60 | 17.32 | 9.38 | 606.54 | 662.6 | 119.4 | 4.2 | 329.8 | 19.6 | 30.4 | 80.0 |
| *weisi* | Worker | 5 | 3.08 | 18.16 | 22..24 | 18.22 | 28.26 | 19.52 | 16.38 | 17.34 | 178.54 | 1182.0 | 244.6 | 14.2 | 676.4 | 51.0 | 316.6 | 91.8 |
| *weisi* | Queen | 5 | 4.39 | 17.64 | 20.08 | 16.28 | 25.56 | 17.46 | 14.26 | 15.64 | 146.84 | 1034.8 | 200.0 | 15.8 | 547.6 | 55.2 | 300.4 | 83.2 |
| *weisi* | Male | 4 | 3.47 | 17.62 | 19.23 | 17.73 | 27.08 | 19.30 | 16.75 | 17.30 | 150.88 | 1197.0 | 237.5 | 17.3 | 662.5 | 59.5 | 312.3 | 86.8 |
| *wilimatae* | Worker | 4 | 5.29 | 16.47 | 14.10 | 17.38 | 23.25 | 17.70 | 16.08 | 16.58 | 110.78 | 2950.0 | 458.0 | 100.5 | 1223.0 | 306.0 | 716.0 | 57.5 |
| *wilimatae* | Queen | 3 | 6.21 | 15.60 | 13.00 | 20.60 | 25.60 | 20.70 | 19.40 | 19.60 | 113.90 | 3534.0 | 530.0 | 128.0 | 1409.0 | 391.0 | 839.0 | 48.0 |
| *wilimatae* | Male | 1 | 4.43 | 19.07 | 17.40 | 7.70 | 16.20 | 8.70 | 6.10 | 7.50 | 101.40 | 1198.0 | 242.0 | 18.0 | 665.0 | 51.0 | 347.0 | 86.0 |
| *wurflenii* | Worker | 5 | 3.94 | 48.62 | 28.68 | 15.70 | 22.34 | 15.98 | -1.30 | 7.22 | 699.48 | 778.6 | 104.6 | 39.2 | 292.2 | 127.2 | 291.0 | 32.4 |
| *wurflenii* | Queen | 2 | 5.40 | 48.65 | 29.00 | 15.70 | 22.15 | 15.70 | -1.95 | 6.75 | 710.65 | 817.0 | 113.0 | 38.0 | 322.0 | 125.0 | 322.0 | 38.5 |
| *wurflenii* | Male | 2 | 3.70 | 46.65 | 31.30 | 15.65 | 23.00 | 16.35 | -2.30 | 7.00 | 754.95 | 821.0 | 117.0 | 37.0 | 329.5 | 122.5 | 326.5 | 41.0 |
| Table 1b. Thorax length (mm) and climatic parameters of cuckoo *Bombus* taxa considered in the comparative analyses | | | | | | | | | | | | | | | | | | |
| *Species* | Sex | n | TW | LAT | TAR | MTWQ1 | MTW | MTWQ2 | MTDQ | AMT | TS | AP | PWM | PDM | PWQ1 | PDQ | PWQ2 | PS |
| *ashtoni* | Male | 5 | 3.49 | 43.54 | 43.24 | 19.96 | 28.80 | 20.36 | -7.58 | 7.24 | 1077.68 | 877.6 | 114.2 | 29.0 | 323.8 | 103.0 | 323.0 | 40.6 |
| *ashtoni* | Female | 5 | 4.54 | 44.99 | 44.30 | 18.42 | 26.66 | 18.42 | -8.84 | 5.14 | 1092.62 | 867.0 | 106.6 | 43.0 | 297.2 | 142.0 | 297.2 | 33.0 |
| *barbutellus* | Male | 3 | 4.00 | 51.37 | 23.20 | 9.17 | 22.17 | 16.70 | 9.67 | 9.53 | 560.23 | 739.3 | 79.3 | 49.0 | 157.3 | 154.0 | 187.7 | 16.7 |
| *barbutellus* | Female | 8 | 4.84 | 50.99 | 26.93 | 12.66 | 21.76 | 15.65 | 2.15 | 7.29 | 655.18 | 829.5 | 102.8 | 45.4 | 265.0 | 147.0 | 275.5 | 26.0 |
| *bohemicus* | Male | 4 | 4.18 | 52.91 | 20.93 | 73.50 | 20.45 | 15.30 | 10.35 | 8.80 | 497.45 | 784.8 | 82.3 | 53.5 | 159.8 | 167.5 | 198.0 | 14.5 |
| *bohemicus* | Female | 9 | 4.41 | 51.38 | 27.39 | 11.11 | 21.26 | 15.16 | 2.24 | 6.52 | 675.17 | 823.4 | 94.6 | 51.2 | 231.8 | 161.4 | 247.4 | 21.4 |
| *campestris* | Male | 3 | 3.69 | 51.94 | 23.47 | 12.27 | 21.37 | 15.80 | 5.10 | 8.47 | 573.70 | 687.7 | 77.7 | 44.3 | 182.7 | 138.3 | 199.7 | 18.7 |
| *campestris* | Female | 2 | 4.96 | 53.49 | 19.40 | 44.00 | 19.90 | 15.00 | 13.50 | 9.00 | 451.70 | 843.0 | 85.0 | 59.0 | 146.0 | 185.0 | 197.0 | 12.0 |
| *citrinus* | Male | 5 | 3.48 | 39.89 | 34.30 | 11.52 | 28.16 | 21.42 | 7.50 | 10.88 | 830.26 | 1143.0 | 114.4 | 77.4 | 321.4 | 247.2 | 286.0 | 11.2 |
| *citrinus* | Female | 5 | 4.84 | 41.74 | 39.58 | 20.64 | 28.68 | 21.04 | -3.52 | 9.12 | 964.24 | 943.0 | 106.0 | 54.8 | 298.4 | 177.6 | 317.8 | 23.6 |
| *fernaldae* | Male | 10 | 3.20 | 39.84 | 32.90 | 80.70 | 25.53 | 16.97 | 7.43 | 8.04 | 698.61 | 766.7 | 125.9 | 12.7 | 349.3 | 50.2 | 119.5 | 63.9 |
| *fernaldae* | Female | 15 | 4.27 | 41.34 | 30.26 | 32.00 | 23.39 | 14.45 | 11.07 | 6.46 | 606.30 | 815.1 | 139.0 | 13.2 | 383.4 | 50.3 | 65.8 | 64.7 |
| *insularis* | Male | 5 | 3.13 | 37.82 | 26.36 | 80.40 | 27.40 | 19.04 | 18.66 | 12.96 | 456.54 | 774.0 | 134.8 | 4.8 | 385.0 | 29.8 | 36.6 | 76.4 |
| *insularis* | Female | 5 | 4.44 | 38.28 | 33.92 | 51.60 | 23.26 | 13.72 | 5.40 | 4.68 | 678.90 | 727.8 | 127.6 | 10.6 | 345.6 | 42.6 | 113.8 | 66.4 |
| *quadricolor* | Male | 5 | 3.82 | 49.90 | 25.98 | 15.60 | 22.68 | 16.52 | 1.80 | 8.54 | 632.32 | 647.2 | 79.0 | 38.8 | 213.6 | 121.2 | 211.2 | 24.2 |
| *quadricolor* | Female | 5 | 4.16 | 51.63 | 24.28 | 13.84 | 21.66 | 15.96 | 3.42 | 8.36 | 598.10 | 656.6 | 76.2 | 41.4 | 190.0 | 129.0 | 200.2 | 20.0 |
| *rupestris* | Male | 5 | 3.51 | 49.01 | 27.88 | 15.50 | 21.68 | 15.50 | -1.00 | 6.84 | 686.76 | 846.0 | 113.6 | 44.2 | 324.0 | 144.6 | 324.0 | 34.8 |
| *rupestris* | Female | 5 | 5.54 | 48.81 | 28.94 | 16.80 | 23.38 | 16.80 | -0.04 | 7.94 | 704.58 | 821.4 | 111.6 | 42.4 | 316.8 | 137.2 | 316.8 | 36.0 |
| *suckleyi* | Male | 3 | 3.58 | 43.91 | 35.17 | 37.30 | 24.70 | 14.60 | 12.07 | 5.10 | 729.43 | 1233.0 | 192.3 | 28.3 | 546.3 | 116.7 | 137.0 | 41.3 |
| *suckleyi* | Female | 5 | 5.21 | 47.92 | 30.58 | -13.40 | 24.24 | 15.54 | 15.08 | 6.50 | 687.14 | 1101.2 | 167.6 | 30.0 | 483.4 | 112.2 | 125.0 | 44.8 |
| *variabilis* | Male | 5 | 3.79 | 33.37 | 31.86 | 25.04 | 32.44 | 25.92 | 10.30 | 17.28 | 704.58 | 1180.4 | 175.8 | 45.4 | 470.2 | 151.0 | 461.0 | 42.6 |
| *variabilis* | Female | 4 | 4.82 | 30.78 | 28.18 | 26.50 | 32.65 | 26.50 | 16.00 | 19.48 | 577.08 | 1274.3 | 180.8 | 59.5 | 494.0 | 196.5 | 494.0 | 36.0 |
| *vestalis* | Male | 2 | 3.55 | 50.09 | 28.00 | 17.60 | 24.00 | 17.60 | -0.30 | 8.70 | 697.50 | 514.0 | 74.0 | 21.0 | 214.0 | 70.0 | 214.0 | 45.0 |
| *vestalis* | Female | 3 | 5.13 | 51.14 | 24.42 | 11.48 | 21.80 | 15.72 | 4.78 | 8.12 | 590.74 | 768.2 | 91.8 | 44.8 | 221.4 | 143.2 | 241.8 | 25.4 |
